# Effect-directed analysis of genotoxicants in food packaging based on HPTLC fractionation, bioassays, and toxicity prediction with machine learning

**DOI:** 10.1007/s00216-024-05632-y

**Published:** 2024-11-23

**Authors:** Alan J. Bergmann, Katarzyna Arturi, Andreas Schönborn, Juliane Hollender, Etiënne L. M. Vermeirssen

**Affiliations:** 1 Swiss Centre for Applied Ecotoxicology, Überlandstrasse 133, 8600 Dübendorf, Switzerland; 2 Eawag Department of Environmental Chemistry, Überlandstrasse 133, 8600 Dübendorf, Switzerland; 3 Zurich University of Applied Sciences, Grüental 14, 8820 Wädenswil, Switzerland; 4 Institute of Biogeochemistry and Pollutant Dynamics, ETH Zürich, 8092 Zurich, Switzerland

**Keywords:** Non-target analysis, Bioassay-guided fractionation, Food contact materials, Non-intentionally added substances, Suspect list, Artificial intelligence

## Abstract

Many chemicals in food packaging can leach as complex mixtures to food, potentially including substances hazardous to consumer health. Detecting and identifying all of the leachable chemicals are impractical with current analytical instrumentation and data processing methods. Therefore, our work aims to expand the analytical toolset for prioritizing and identifying chemical hazards in food packaging. We used a high-performance thin-layer chromatography (HPTLC)-based bioassay to detect genotoxic fractions in paperboard packaging. These fractions were then processed with non-targeted liquid chromatography high-resolution mass spectrometry (LC-HRMS/MS) and machine learning-based toxicity prediction (MLinvitroTox). The HPTLC bioassay detected four genotoxic zones in extracts of the paperboard. One-dimensional HPTLC separation and targeted fraction collection reduced the number of chemical features extracted from paperboard and detected with LC-HRMS by at least 98% (from 1695–2693 to 14–50). The entire process was successful for spiked genotoxic chemicals, which were correctly prioritized in the fractionation and non-target analysis workflow. The native chemical with the strongest genotoxicity signal was identified with a suspect list as 5-chloro-2-methyl-4-isothiazolin-3-one and confirmed with LC-HRMS/MS and HPTLC bioassay. Toward identification of the remaining unknown genotoxicants, two-dimensional HPTLC further reduced the number of chemical features. Genotoxicity predictions with MLinvitroTox based on molecular fingerprints of the unknown signals derived from their MS2 fragmentation spectra helped prioritize two chemical features and suggested candidate structures. This work demonstrates strategies for using HPTLC, HRMS, and toxicity prediction to help identify toxicants in food packaging.

**Graphical Abstract:**

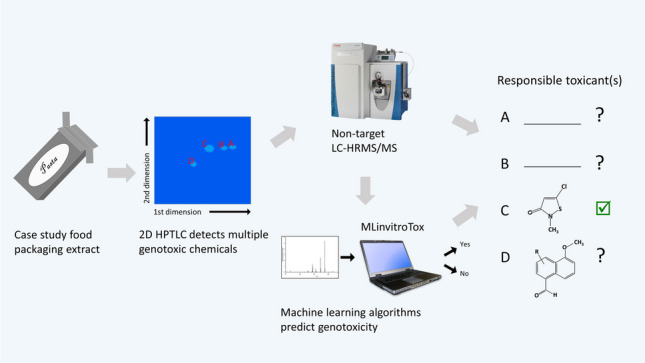

**Supplementary Information:**

The online version contains supplementary material available at 10.1007/s00216-024-05632-y.

## Introduction

Packaging protects food from external contamination and maintains freshness during transport. However, packaging materials typically contain many chemicals which could migrate to food, potentially impacting consumer health. Chemicals in packaging can include ingredients, which are intentionally added to packaging and appropriately regulated [1]. Chemicals that are inadvertently added to packaging may also be present, for example, due to impurities of ingredients, reaction by-products, or other contamination [2]. Such non-intentionally added substances (NIAS) almost certainly occur as complex mixtures including unknown chemicals. NIAS are regulated in the European Union in that no chemical may migrate to food at levels that could harm human health [3]. Whereas known packaging ingredients can be monitored with targeted chemical analysis, the hazard and concentration of unknown NIAS are not easy to assess. Therefore, enhanced methods are needed to detect and identify potentially harmful substances in food packaging, enabling more robust risk assessment.

There can be many hundreds of chemicals in food packaging, of which only a minority may be harmful. Identifying chemicals in a mixture with state-of-the-art analytical tools can be prohibitively resource-intensive and time-consuming [4]. Attempting to identify all chemicals in food packaging, even when focusing efforts solely on chemicals leaching to food, is not feasible with current tools. Identification efforts should instead be focused on chemicals that pose the greatest concern for consumer health. To that end, bioassays can help direct investigators to chemicals with specific toxicity [5], and modern chemical analysis and in silico tools can prioritize chemical candidates most likely to be responsible for toxicity [6].

One of the most concerning hazards for human health is genotoxicity, which is apparent in risk assessment of NIAS following the threshold of toxicological concern (TTC) concept: chemicals with structural alerts for genotoxicity would only be allowed in the diet at amounts 600 times lower (0.0025 µg/kg b.w./day) than the next most concerning level (class III, 1.5 µg/kg b.w./day) [7, 8]. Genotoxic chemicals are relevant because they have been observed in food packaging, both with chemical analysis and bioassays. Using suspect screening and non-target analysis, Rusko et al. found chemicals that were predicted to be mutagenic and carcinogenic migrating from paper straws into food simulants [6]. Ozaki et al. observed in vitro genotoxicity in 46% (13/28) of paper packaging extracted with ethanol and attributed some of the effects to 1,2-benzisothiazoline-3-one, but could not explain the genotoxicity for a majority of samples [9]. Rosenmai et al. detected genotoxicity in eight of 20 extracts of paper products with p53 CALUX and *Salmonella* reverse mutation tests, but they did not investigate the cause of toxicity [10].

We recently evaluated an in vitro bioassay for direct-acting genotoxicity on high-performance thin-layer chromatography (HPTLC) plates [11]. The assay first separates chemicals on a silica gel HPTLC plate, and then detects separated chemicals with a bacterial reporter gene assay for DNA repair response, the umuC SOS test directly on the plate [12, 13]. Compared to bioassays of whole mixtures, HPTLC bioassays provide information about the minimum number of bioactive chemicals in a sample and can separate them from potentially interfering (e.g., cytotoxic) matrix components [11, 14]. The HPTLC format is also sensitive to low levels of chemicals compared to microtiter bioassays [11, 14]. However, as the potencies of different chemicals can vary by orders of magnitude, absence of toxicity in migrates of food packaging — even with a sensitive HPTLC assay format — would not ensure safety for chemicals of low genotoxic potency [15]. Worst-case studies using concentrated sample extracts are more likely to capture potential hazards [16]. Identified chemicals in such extracts could then be evaluated with thorough risk assessment and monitored with targeted chemical analysis in food simulant migrates.

HPTLC allows for sample fractionation and thus suits effect-directed analysis (EDA) strategies [17–19] with fraction collection and subsequent compound identification targeting zones that show toxicity. Meyers et al. used HPTLC-umuC and direct-injection HRMS to investigate genotoxicants in food packaging. They identified and confirmed the toxicity of fatty acid epoxides while several other genotoxic chemicals remained unidentified [20]. HPTLC typically separates chemicals over a short distance of about six cm in one direction, so peak capacity is limited. Applying a second dimension of separation (2D chromatography) [21, 22] can improve resolution but is not guaranteed to isolate unknown toxicants. Several chemicals may still be co-retained on an HPTLC plate or overlap enough to co-elute with the zone of interest. Further separation of fractions with LC-HRMS and acquisition of MS2 spectra support identification. Stütz et al. substantially reduced the number of suspect acetylcholinesterase inhibitors in water samples using 2D techniques involving HPTLC separation, fraction elution, and reapplying to HPTLC for a second chromatography step. They ultimately identified some responsible chemicals but most active substances were not identified [23]. Identification of active substances will be most difficult for relatively potent toxicants present at low abundance, even in a 2D fraction with a simplified mixture of chemicals. Separation with 2D chromatography on the same plate, instead of eluting and reapplying fractions, could improve recoveries and therefore the chance of detecting chemicals of lower abundances.

Detected chemicals can also be filtered by toxicological information, even if they have not been assigned final structures. Unidentified LC-HRMS/MS features, defined here as paired retention time, exact mass-to-charge ratio, and MS2 spectrum, contain structural information that can be used in quantitative structure–activity relationships. Such models apply molecular substructures, descriptors, or fingerprints as input to predict bioactivity for chemicals with known structures that have not yet been toxicologically evaluated. For unidentified HRMS/MS signals, although we lack structures to compute molecular fingerprints, structural clues can be obtained from the fragmentation spectra (MS2). In particular, advances in machine learning tools have enabled the deduction of structural information from HRMS/MS data via molecular fingerprints [24]. These fingerprints are mathematical representations of two-dimensional chemical structures encoded as binary strings of 0’s and 1’s representing in each bit the absence or presence, respectively, of specific molecular substructures. The most likely candidates for a specific LC-HRMS/MS feature can be found by comparing its predicted fingerprints with fingerprints of known compounds. Arturi et al. used this strategy of deriving molecular fingerprints from MS2 spectra to develop MLinvitroTox, which classifies unidentified HRMS/MS features as active/inactive according to more than 500 in vitro endpoints, such as endocrine disruption and genotoxicity [25].

In the current work, we explored prioritizing unknown chemicals for identification by combining 1D and 2D HPTLC fractionation with a genotoxicity bioassay, analyzing active zones with LC-HRMS/MS, and applying machine learning-based toxicity prediction from MLinvitroTox to the experimental results. We expected that HPTLC fractionation would substantially reduce chemical features and that MLinvitroTox would help further prioritize unknown features for potential genotoxicity. We evaluated the workflow for spiked genotoxicants in a “mock” EDA. We then used the approach to investigate the cause of genotoxicity in a case study extract of paperboard, building on bioactivity observations in our previous publication [11]. This paper describes progress made toward identifying toxicants in food packaging and supports development of HPTLC-based EDA for diverse sample types.

## Materials and methods

### Sampling

The generation of paperboard extracts and their initial screening in HPTLC-umuC are described in Bergmann et al. [11]. Anonymized samples of printed and recycled paperboard that never had been in contact with food (*n* = 1 for each sample type) were extracted with organic solvents as follows. The samples were cut into pieces of 1–2 cm^2^, and placed in 45 mL methanol:hexane:methyl-*t*-butyl ether 20:20:5 for 18 h at room temperature. The extracts were subsequently filtered through a folded paper filter and concentrated to 10 mL with a Rotavapor at 55 °C and 500 mbar to a final concentration of 0.4 g paperboard/mL. Process controls following the same procedure confirmed that no genotoxicity was due to extraction procedures.

### HPTLC-umuC

HPTLC-umuC was performed according to Bergmann et al. [11]. For further details, see also Text S1. Standard chemicals and paperboard extracts were applied to pre-washed silica gel 60 HPTLC plates in 6-mm bands with an Automated TLC Sampler 4 (ATS4, CAMAG). Chromatographic development was performed with an Automated Multiple Development 2 (AMD2, CAMAG) as described in the “EDA strategy” section.

Bacteria for the umuC assay, *Salmonella typhimurium* TA1535 psk1002, were obtained from German Collection for Microorganisms and Cell Cultures (DSMZ, Braunschweig, Germany) and cultivated overnight before a test. A Derivatizer (CAMAG) was used to spray 3 mL bacteria to a prepared HPTLC plate, which was incubated for 2 h. After incubation, the HPTLC plate was dried for approximately 5 min with a hair dryer. The indicator chemical, 4-methylumbelliferyl-β-D-galactoside, was sprayed onto the HPTLC plate, which was then incubated for 0.5 h. The HPTLC plate was removed and dried again. Finally, the HPTLC plate was exposed to NH_3_ vapor in a twin trough chamber. Images of HPTLC plates were collected after every step with a Visualizer 2 (CAMAG). After the final step, the HPTLC plate tracks were scanned for fluorescence with the Scanner 3 (CAMAG).

### EDA strategy

#### One-dimensional chromatography

Coordinates of bioactive zones were determined by applying a sample, and bioassay negative and positive controls, to two sides of a 20 × 10 cm HPTLC plate (Fig. S1). One “bioassay side” was intended for determining the retention factors of bioactive zones with the umuC bioassay, and the other, “MS side,” for collecting fractions without bioassay for LC-HRMS/MS analysis. On the MS side, samples were applied in triplicate 6-mm bands with 50 µL sample per band to increase sample mass on the plate without overloading the silica. After optimization of chromatography (Text S2), chromatography was performed with AMD2 consisting of 100% methanol to 20 mm, 100% ethyl acetate to 35 mm, 66.6:33.4 ethyl acetate:n-hexane to 55 mm, and 50:50 ethyl acetate:n-hexane to 80 mm. The developed HPTLC plate was cut by scoring the glass with a TLC plate cutter (CAMAG). The HPTLC-umuC was performed on the “bioassay side.” The coordinates of bioactive zones were determined with fluorescence images from the Visualizer 2.

Following a scheme outlined in Fig. S1, a “mock” EDA was performed with recycled paperboard spiked with 4-nitroquinoline 1-oxide (4-NQO) and 5-chloro-2-methyl-4-isothiazolin-3-one (CMIT) was investigated with one-dimensional (1D) HPTLC-EDA as if it was a native sample. Fraction elution was guided by the bioactivity retention factors detected on a parallel plate. Fractionation recovery was assessed before chemical analysis. Non-target analysis workflows in Compound Discoverer were applied to prioritize LC-HRMS peaks.

#### Two-dimensional chromatography

Two-dimensional (2D) chromatography was used for native unknown genotoxicants in a printed paperboard extract. A scheme of the process is shown in Fig. S2. Printed paperboard extract was applied (ATS4, CAMAG) to two 10 × 10 cm HPTLC plates with the pre-wash solvent front positioned to the left using 150 µL in 18-mm-long bands 10 mm from the bottoms and 10 mm from the right edges of the plates. The plates were placed side-by-side in the AMD2, leaving a gap between the plates to avoid capillary effects on the inner edges. The first dimension of chromatography was performed in the *Y*-direction with the same method as 1D chromatography. The plates were removed from the AMD2, documented with Visualizer 2, and placed again in the AMD2, each rotated 90° clockwise. The second chromatography step was 100% methanol to 30 mm, and then 20:80 ethanol:n-hexane to 70 mm. Ethanol was chosen as the second dimension separation solvent based on its different selectivity to the first dimension, ethyl acetate [26], and optimized with trial separations of the sample. HPTLC-umuC was used to determine the 2D coordinates of bioactive zones on one of the parallel plates. The positive control, 4-NQO, was applied as a bioassay control after both chromatography steps.

#### Fraction elution

TLC-MS Interface 2 (CAMAG) was connected to an HPLC pump (Bischoff model 2200) and 5 bar nitrogen. The elution head of the TLC-MS Interface 2 was positioned relative to the bioactive zones by manually aligning the HPTLC plate with the rulers on the device platform. Either triplicate (1D chromatography) or single (2D chromatography) zones were stamped with a 2 × 4 mm oval elution head and eluted into a single amber chromatography vial with 0.2 mL/min methanol for 1 min. Blank zones were collected first at the desired retention factor, but in the bioassay negative control track. These blank cuts were used to check the alignment of the TLC-MS interface. Fractions of the samples were then collected from the center of the zone, and directly above and below, leaving an intact silica gap of approximately 1 mm.

Manual collection of fractions was based on coordinates for a rectangular extraction boundary derived from bioactive zones in a HPTLC-umuC. Silica was then extracted from a parallel HPTLC plate that had not been used for bioassay. The silica was extracted by cutting a border in the silica with a scalpel and scraping the inner silica with a flat metal spatula onto folded piece of aluminum foil. The loose silica was guided into an amber glass chromatography vial to which 600 µL methanol or ethyl acetate was then added. After 10 min of sonication at room temperature, the entire contents were filtered through a 0.45-µm PTFE filter into a new amber glass vial. The process was repeated to collect an outer fraction, consisting of the silica within 1-mm circumference of the already extracted main inner fraction.

#### Confirmation of fractionation recovery

For plates extracted with the TLC-MS interface and manual collection, the HPTLC-umuC was performed on the now-extracted HPTLC plates. Success of fraction collection was evaluated by reapplying 100 µL of the fractions (25 µL parent sample equivalent) in 4-mm bands to an HPTLC plate, performing 1D chromatography, and the HPTLC-umuC.

### LC-HRMS/MS

Aliquots of parent (unfractionated) samples, fractions, blanks, and standards in methanol were spiked with internal standards (Table S1) [27, 28]. They were exchanged to 10% methanol:90% water v/v by evaporating to dryness under a gentle nitrogen stream on a heating block set to 40 °C. The samples were quickly reconstituted in 10% methanol. The nominal concentrations of fractions and parent samples were normalized at this step. Specifically, fractions were four times diluted during fractionation so 400 µL of a fraction was exchanged to 200 µL 10% methanol (final dilution factor = 2) and 100 µL parent extract was exchanged to 200 µL 10% methanol (final dilution factor = 2).

Liquid chromatography was performed based on methods described previously [27], and with minor adaptations for representative target chemicals (Text S3). 20 µL injections were made in triplicate and were preceded by a single injection of blank 10% methanol. Separation was performed on a C18 column (Atlantis T3 3 × 150 mm, 3 µm pore size, Waters) at 30 °C. The mobile phase starting condition was 100% 18 MΩ water with 0.1% formic acid (solvent A), 0% HPLC grade methanol with 0.1% formic acid (solvent B). The solvent gradient (Dionex UltiMate3400 RS pump, Thermo Scientific) proceeded at 0.3 mL/min from 0% B to 95% B from 1.5 to 18 min, then held at 95% B for 10 min before returning to starting conditions and equilibrating for 4 min.

High-resolution mass spectrometry was performed with a QExactive Plus Orbitrap (Thermo Scientific) with electrospray ionization in positive mode, spray voltage at 4 kV. Full scans were acquired from 100–1000 m/z with a resolution of 140,000. Five MS2 in each scan were triggered at a resolution of 17,500 by a list of 378 masses corresponding to known genotoxicants and migrants of food packaging [11], or the most abundant masses. For cases in which MS2 were still not collected for prioritized chemical features, the unfractionated paperboard extract was reanalyzed with LC-HRMS/MS using a mass list of the prioritized features as an inclusion list for triggering MS2 fragmentation. This was used to obtain missing MS2 for three of the 13 prioritized features for zone D.

### LC-HRMS/MS data analysis

HRMS data of parent samples, fractions, and blanks were processed with Compound Discoverer (Ver. 3.2, Thermo Scientific). Retention times (RT) were aligned and peaks were detected at an abundance threshold of 1 × 10^6^, and features common to multiple samples were merged within a mass error of 2 ppm. Gaps were filled with a signal to noise threshold of three. Features were annotated with compounds from mzCloud, Chemspider, a custom suspect list of genotoxicants and migrants containing 378 substances [11]. More Compound Discoverer settings are given in Table S2.

Chemical features that were detected by the peak-picking workflow were filtered on their presence in fractions of interest. Specifically, chemical features must have been above the abundance threshold of 1 × 10^6^ (i.e., no filled gaps) in the parent samples because the abundance was expected to be highest in the unfractionated sample. The ratio of peak abundances of grouped injection triplicates of the fractions must have been between 10 and 75% of the parent samples, and above 10 times greater than any peak detected in blank HPTLC plate fractions. Finally, a peak must not have been detected in any fraction not associated with the zone of interest.

Representative MS2 spectra of prioritized features were selected from Compound Discoverer or manually from the raw acquisition file, and then exported to RAW from Freestyle 1.3 (Thermo Scientific). The RAW MS2 spectra were converted to .mgf using MSConvert 3.0 (Proteowizard). MS2 spectra were then imported into SIRIUS (v. 5.6.2) [24], to predict the molecular formula as a cross check of Compound Discover, generate predicted molecular fingerprints from MS2 [29], and compare them to chemical fingerprints derived from structures in publicly available chemical libraries (Fig. S3 and Table S3). The similarities of the structure fingerprints to the MS2-based fingerprints of the unknowns were ranked by SIRIUS with the CSI.fingerIDScore [29].

### Toxicity prediction using machine learning

The molecular fingerprints generated by SIRIUS for each individual chemical feature were evaluated for potential genotoxicity with MLinvitroTox [25]. Genotoxicity models were built on data available in the Integrated Chemical Environment (ICE) database from the Tox21 project [25, 30]. Bioassays in ICE were selected from two general modes of action: DNA damage and p53 signaling pathway. The initial data were curated according to the ICE guidelines, removing samples associated with warning flags for the assays or chemicals. For each mode of action, we selected bioassays with the most data entries and therefore assumed to be the most robust for model training. The selected bioassays were p53_bla_ratio for p53 activation (5953 chemicals tested with 723 active) and three DT40 cell bioassays for DNA damage (3057 chemicals tested with 257 active). Data from p53_bla bioassays listed with different protocol numbers were combined to train a single model. For the DT40 DNA damage assays, we refined the ICE data to capture the genotoxicity metric of this assay. We implemented ratios of target endpoints (DT40_100 and DT40_657) to control (DT40_wt) [31]. Structures of chemicals in the final toxicity datasets were cleaned according to the logic in Arturi et al. [25], and SIRIUS fingerprints were computed using in-house developed scripts. Structural molecular components and toxicity targets were used as inputs to XGBoost algorithms to train supervised classifiers predicting genotoxicity from molecular fingerprints as described in detail by Arturi et al. [25]. The models for p53 activation and DNA damage were trained with 0.85/0.15 train/test splits. Further details of the calibration and evaluation of MLinvitroTox models are given in Text S4 and Table S4. Molecular fingerprints were considered potentially genotoxic if they were more likely than not (*P* > 0.5) to be active in at least one genotoxicity model. As a check of the modeling, a set of known genotoxic chemicals were submitted to MLinvitroTox with a majority (10/13) predicted to be active in at least one endpoint (Table S5). We also analyzed genotoxicity prediction success on the molecular fingerprints predicted from measured MS2 data of the spiked and native known genotoxic chemicals, 4-NQO and CMIT, collected during fractionation experiments. Each of five available MS2 spectra was correctly predicted active in at least one endpoint (Table S6).

### Confirmation of toxicants

Tentatively identified toxicants were obtained and confirmed with chemical analysis and HPTLC-umuC. They were analyzed for RT, m/z, and fragmentation with LC-HRMS/MS. They were evaluated for bioactivity and retention factor with HPTLC-umuC. Any mismatch in parameters deconfirmed candidate toxicants.

## Results and discussion

### One-dimensional EDA: CMIT a culprit

The goal of performing a mock EDA was to check that through the process of HPTLC-umuC zone selection, fraction elution, chemical analysis, and non-target peak prioritization, we would successfully recover, detect, and prioritize the correct substances. Recycled paperboard had the highest apparent matrix following HPTLC-umuC so was expected to be a challenging sample for a mock EDA. 4-NQO is a common positive control in genotoxicity assays and one of the most potent in HPTLC-umuC [11]. Therefore, we chose 4-NQO because it would challenge the chemical analysis to detect low levels of potent genotoxicants. CMIT was selected because it is a food contact chemical that is also relatively potent in umuC [11]. The unfractionated recycled paperboard extract with the two spiked genotoxic compounds contained 1695 chemical features. 1D HPTLC fractionation and non-target analysis workflow reduced the chemical features to 14 and 27 in bioactive zones targeting the spiked 4-NQO and CMIT, respectively (Table 1). Both 4-NQO (Fig. S4) and CMIT were among the prioritized features, indicating a successful workflow.

**Table 1:**
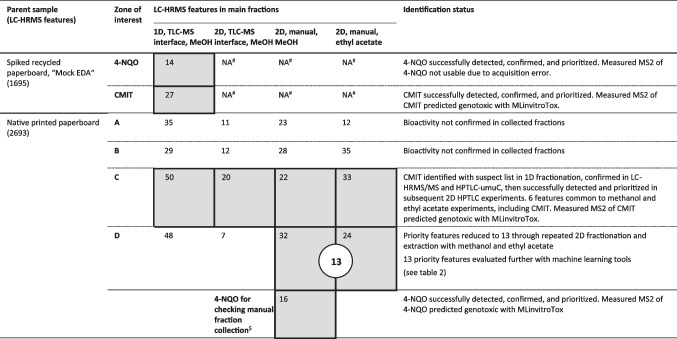
Summary of HPTLC-EDA experiments and their outcomes. Chemical features (intensity > 10^6^) remaining after HPTLC fractionation and prioritization LC-HRMS non-target workflows are shown for two spiked chemicals and four unknown bioactive chemicals. Gray shading indicates fractions in which bioactivity was confirmed through reapplication to HPTLC-umuC

^#^*NA* not applicable. CMIT was a native component of printed paperboard, so it was not spiked in 2D fractionation experiments. Instead, the native feature identified as CMIT was used as a control in 2D experiments. ^$^4-NQO was evaluated to control the manual extraction process, in addition to the identified known zone C toxicant, CMIT. 4-NQO was applied as a bioassay control after HPTLC-chromatography, then collected with manual extraction. Therefore, the prioritization filters were different than other fractions in that the fraction was only compared to blank HPTLC fractions. *1D* one-dimensional chromatography, *2D* two-dimensional chromatography, *MeOH* methanol

**Fig. 1:**
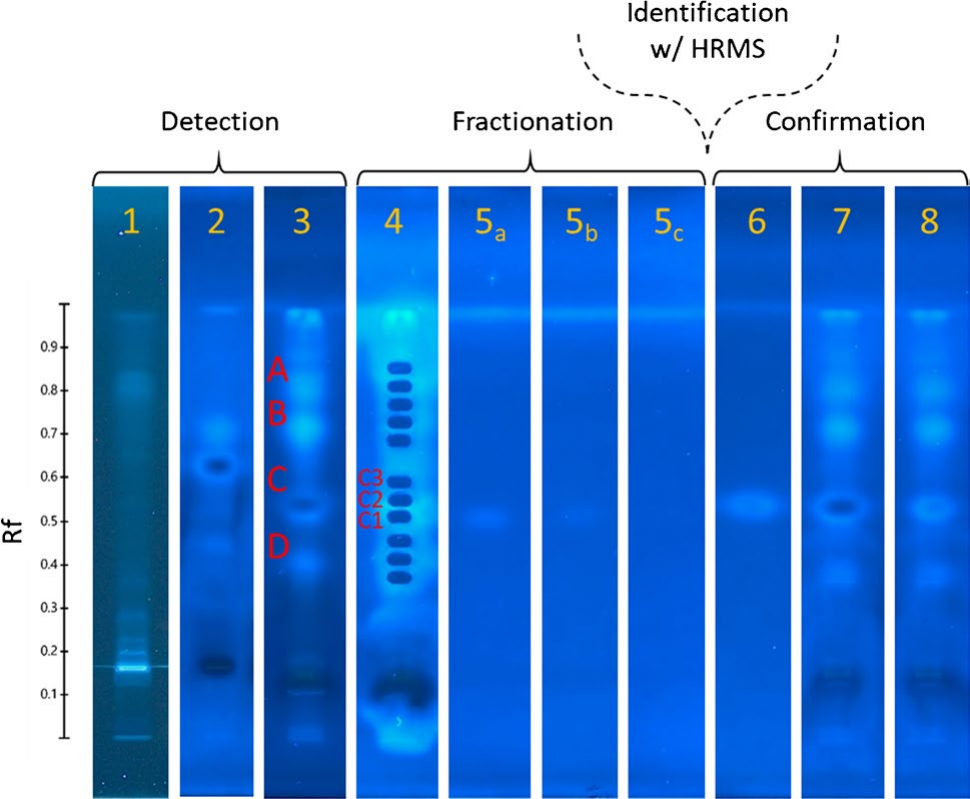
Example of workflow for 1D EDA with HPTLC-umuC showing the steps from detection of bioactivity through fractionation, identification, and confirmation of tentatively identified chemicals. Tracks 1 + 2: 20 µL unspiked printed paperboard extract developed with isocratic separation (50:50 acetone:n-hexane) before (track 1) and after (track 2) bioassay. Track 3: 50 µL printed paperboard extract separated with optimized chromatography (ethyl acetate-based multiple development, see main text). This track was used to determine retention factors of active substances for fraction elution. Track 4: After fraction elution and subsequent bioassay on remaining track. Tracks 5a–5c: Fractions C1–C3 (left to right), reapplied for fractionation confirmation in HPTLC-umuC. Track 6: CMIT standard (5 ng). Track 7: 20 µL printed paperboard extract spiked with 5 ng CMIT. Track 8: 20 µL printed paperboard extract for side-by-side comparison with spiked sample

For unknown substances in printed paperboard extract, optimized separation with ethyl acetate-based solvent mixtures (Text S3) revealed four genotoxic zones (Fig. 1, track 3). The bioassay after fraction elution (Fig. 1, track 4) showed that some bioactivity remained in zone C, centered between fractions C1 and C2. Bioactivity was confirmed in the eluted fractions C1 and C2 at the same retention factor as zone C, with greater intensity in fraction C1 (Fig. 1, track 5a) than in C2, but less than the parent sample. These observations confirmed that the responsible genotoxicant was in fractions C1 and C2 and informed us that the recovery compared to the parent sample was less than 100%.

Fraction C1, which had the greatest intensity of bioactivity, resulted in 50 prioritized LC-HRMS chemical features. One mass matched the entry in our suspect list for CMIT, which had also been used for the mock EDA, and followed the trend observed in the fractionation confirmation (C1 < C2 < C3). This feature was the third most abundant of the prioritized features for fraction C1 (area about 1 × 10^9^ in unfractionated sample). Indeed, comparison to the standard confirmed the chemical identity (Rt, m/z, MS2) of CMIT (Fig. S5). CMIT was bioactive in the HPTLC-umuC at a retention factor matching the unknown in printed paperboard (Fig. 1, track 6). No shoulder peaks were apparent in the overspiked sample (Fig. 1, track 7), indicating that CMIT was the genotoxicant responsible for the strongest bioactive zone in printed paperboard.

CMIT is possibly in the sample as an intentionally added substance in the paperboard manufacturing process. CMIT is added to paperboard fiber, for example, as a biocide in mixture with 2-methyl-4-isothiazolin-3-one in ratio 3:1 sold as Kathon CG [32]. Kathon CG has been known since the 1980s to have mutagenic potential but was not considered to be a hazard and has been even approved for dermatological use [32]. Specifically, Kathon CG induced mutations in a *Salmonella* reverse mutation assay (strain TA100) without metabolic activation. The addition of rat liver S9 eliminated or reduced mutagenic activity of Kathon CG [33].

In a previous study, CMIT was tentatively identified (level 2ab, with MS2 library and in silico fragmentation matches) in migrates of paper straws into 3% acetic acid or 50% ethanol [6]. Mostly with the use of an OECD QSAR Toolbox, CMIT in paper straws was then considered a suspected mutagen but not a suspected carcinogen [6]. According to the Food Contact Chemicals database, CMIT appears in 26 of 67 legislative or industrial chemical lists, including multiple specific to paperboard [34]. Regulations in Germany restrict the total amount of CMIT and related isothiazolinones to 0.5 µg/dm^2^ in extracts of paperboard products [35].

Besides CMIT, three zones remaining in the printed paperboard extract had 35 (zone A), 29 (zone B), and 48 (zone D) prioritized features, but no clear suspect hits as with zone C. To reduce the effort in structure elucidation by further reducing the number of features, we investigated incorporating a second chromatographic dimension in the HPTLC fractionation.

### Two-dimensional EDA: further reduction in features

**Fig. 2:**
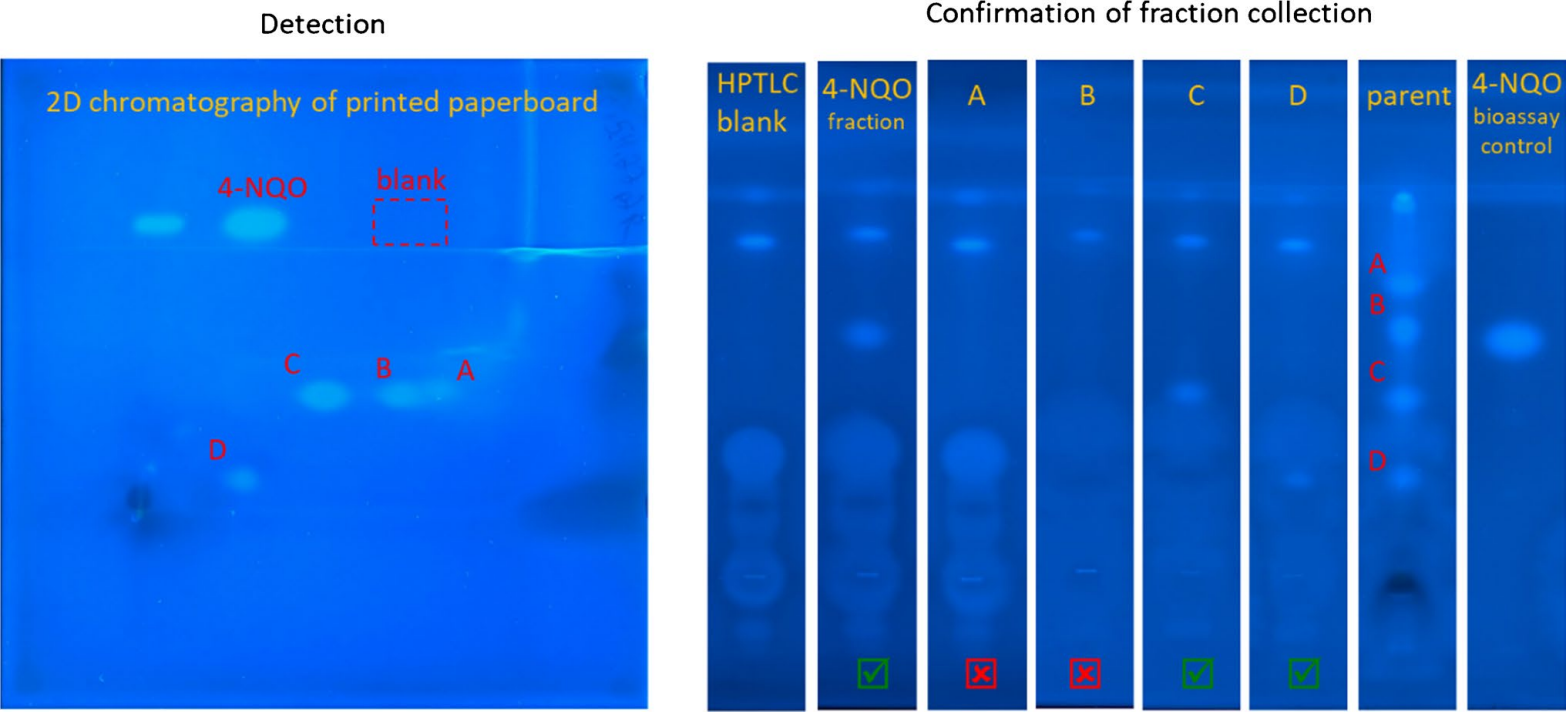
Manual extraction of 2D HPTLC zones of printed paperboard. “Detection” shows the bioassay determining coordinates of bioactive zones A–D. 4-NQO is a bioassay positive control applied to the plate after chromatographic separation at two concentrations. 4-NQO and a blank zone, “HPTLC blank,” were collected as controls for the fractionation process. “Confirmation of fraction collection” shows fractions A–D retested in HPTLC-umuC using 1D chromatography. Bioactivity was successfully recovered for 4-NQO, and zones C and D (green checkmark). Bioactivity was not recovered for zones A and B (red X). The HPTLC blank indicates some background signals were introduced during the manual extraction process, but these did not interfere with detecting fraction bioactivity

As shown in Fig. 2, we performed a second dimension of chromatography by turning a 10 × 10 cm HPTLC plate 90° [36], rather than extracting the fraction and reapplying to a new plate [23]. The ethanolic mobile phase used as a second chromatographic dimension further separated mixture components from each other. This was first evidenced by natively fluorescent chemicals that were co-retained in the first dimension but separated in the second dimension. Bioactive zones A–D were also separated from natively fluorescent chemicals (Fig. S6) and therefore are expected to be separated from undetected chemicals as well. Zone C, the strongest bioactive signal in the sample eluted with TLC-MS Interface, contained 20 chemical features. This was an additional reduction from 50 features in 1D chromatography. CMIT, which was identified previously during 1D chromatography experiments, was one of the 20 features, indicating the successful recovery and prioritization of the correct substance with 2D HPTLC-EDA. In summary, HPTLC fractionation reduced the number of features by 98.1% using 1D chromatography and by a further 75% (20/2693 = 99.3% total) with 2D HPTLC while successfully capturing CMIT among prioritized chemical features (Table 1).

The bioactivity of the zone C fraction (following 2D-HPTLC and TLC-MS Interface elution) was also confirmed by reapplying it in the HPTLC-umuC. However, bioactivity was not detected following TLC-MS Interface elution for zones A, B, and D (Table 1). Therefore, although 2D HPTLC-EDA reduced the number of prioritized features from 35, 29, and 48 to 11, 12, and 7 in zones A, B, and D, we could not be confident that the responsible toxicant was among them. The abundance of CMIT in zone C fraction was about 10% of the unfractionated paperboard extract. If such a recovery of about 10% is also true for the unknown toxicants of zones A, B, and D, they might be below the detection limit of HPTLC-umuC, LC–MS, or both. Stütz et al. [23] observed a similar reduction in features using 2D HPTLC chromatography. Their methods were different in that they used multiple extractions and reapplication to HPTLC plates to perform 2D chromatography and they did not confirm if the final 2D fractions were still bioactive. They noted that a small elution head of the TLC-MS interface and manually aligning the device could lead to low recoveries of extracted chemicals [23]. To better target the unknown toxicants in zones A, B, and D, we attempted manual extraction of the silica with a scalpel and spatula instead of the TLC-MS Interface (Fig. 2, Fig. S7). Manual extraction of zone C resulted in CMIT having 38% abundance by peak area, compared to the unfractionated sample, indicating greater mass recovery than with the TLC-MS interface. However, manual extraction also captured a greater number of background chemicals. Zone C after manual extraction had 22 features compared to 20 with the TLC-MS interface while still containing CMIT. More markedly, the number of prioritized features for zone D increased from seven with the TLC-MS interface to 32 with manual extraction. The recovery of the responsible genotoxicant in zone D could be confirmed by retesting the manually extracted fraction in the HPTLC-umuC (Fig. 2). The recovery of bioactivity for zones A and B (23 and 28 features, respectively) could still not be confirmed. The positive control, 4-NQO, was confirmed in all fractionation experiments.

Although methanol is a strong solvent on normal-phase HPTLC plates, it may not be the best eluent for all chemicals. Zones A and B were retained at high retention factor in the first dimension of chromatography, clearly mobile in an ethyl acetate step. We therefore repeated 2D-HPTLC fractionation except we used ethyl acetate as an extraction solvent of manually collected silica. Again, no bioactivity in collected fractions of zones A and B could be confirmed. Zones A and B were therefore not investigated further.

Both manual ethyl acetate and methanol fractions of zone D after 2D separation did have confirmed bioactivity (Fig. S8), allowing us to further investigate the unknown toxicant(s) in zone D. In fractions of zone D, we detected 32 and 24 chemical features with methanol and ethyl acetate extraction, respectively. Thirteen features were common to these manual methanol and ethyl acetate extracts (Table 2). One of the 13 prioritized features (C_14_H_13_ClO_4_) was also detected in the TLC-MS interface fraction when lowering the Compound Discoverer filter settings to a fraction/parent ratio of 0.05. That it was consistently detected initially made C_14_H_13_ClO_4_ an interesting chemical. However, C_14_H_13_ClO_4_ was the least abundant prioritized feature based on peak area in the unfractionated extract, so evidence for this compound as a responsible toxicant remained weak.

**Table 2:** Prioritized features of zone D

Feature number	Calc. MW (g/mol)	m/z [M + H] +	RT (min)	Area (parent extract)	Predicted molecular formula	Mass error (ppm)	Bioassay in which fingerprint from MS2 predicted to be genotoxic	# Total candidates (SIRIUS)	# Total structures predicted genotoxic (percent)	# Top 10% of structures predicted genotoxic (percent)
1	139.0633	140.07058	9.71	3.9 × 10^7^	C_7_ H_9_ N O_2_	− 0.17	–	2116	18 (0.9)	0 (0)
2^+^	186.0528	187.06011	10.61	1.5 × 10^7^	C_8_ H_10_ O_5_	0.05	–	803	23 (2.9)	3 (3.7)
3^+^	214.0478	215.05504	10.61	9.0 × 10^7^	C_9_ H_10_ O_6_	0.14	–	293	6 (2.0)	0 (0)
4^+^	246.0740	247.08126	10.61	6.8 × 10^7^	C_10_ H_14_ O_7_	0.08	–	233	8 (3.4)	0 (0)
5^+^	232.0583	233.06561	10.61	2.0 × 10^7^	C_9_ H_12_ O_7_	0.12	–	151	4 (2.6)	0 (0)
6^+^	200.0321	201.03933	10.61	2.1 × 10^7^	C_8_ H_8_ O_6_	− 0.17	–	293	8 (2.7)	1 (3.4)
7	263.1005	264.10776	10.81	6.3 × 10^6^	C_10_ H_17_ N O_7_	− 0.08	TOX21DT40ratioup	138	6 (4.3)	1 (7.2)
8	280.0503	281.05753	14.58	6.6 × 10^6^	C_14_ H_13_ Cl O_4_	0.05	–	329	24 (7.3)	0 (0)
9	246.0892	247.09648	15.01	4.6 × 10^7^	C_14_ H_14_ O_4_	− 0.04	–	2757	238 (8.6)	10 (3.6)
10	210.0529	211.06016	15.23	2.1 × 10^7^	C_10_ H_10_ O_5_	0.21	–	1283	41 (3.2)	1 (0.8)
11	150.0318	151.03905	15.23	1.4 × 10^7^	C_8_ H_6_ O_3_	0.54	–	311	10 (3.2)	6 (19)
12^$^	405.1788	406.18602	15.39	3.0 × 10^7^	C_21_ H_27_ N O_7_	− 0.02	TOX21p53BLAup	356	20 (5.6)	2 (5.6)
13^$^	186.0681	187.07534	15.39	2.3 × 10^7^	C_12_ H_10_ O_2_	− 0.07	TOX21DT40ratioup	1085	39 (3.6)	17 (16)

^+^Linked by possible in-source ionization because these features share RT and share common MS2 fragment ions (85.0283, 99.0440, 127.0389). See Fig. S9. ^$^Linked by possible in-source ionization. 187.0753 ([M + H]^+^) is a MS2 fragment of 405.1788. See Fig. S10

Manual evaluation of the 13 prioritized features revealed that some share retention time and share m/z in their MS2 spectra, indicative of potential in-source ionization (Text S5) [37]. Therefore, it was deduced that the 13 features actually represent only eight chemicals. For modeling purposes, we treated each feature independently, but considered possible in source fragmentation while interpreting the results.

### Structure and toxicity prediction of prioritized candidates

Table 2 shows the 13 prioritized features for zone D and the results of structure and toxicity prediction. Three of the 13 prioritized features’ predicted molecular fingerprints were potentially active (*P* > 0.5) in at least one genotoxicity bioassay. Features 7 (C_10_H_17_NO_7_), 12 (C_21_H_27_NO_7_), and 13 (C_12_H_10_O_2_) were each predicted to be active in one assay. Features 12 and 13 are notable because they are linked by LC-HRMS/MS data as possible in-source fragmentation products (Text S5 and Figs. S8 and S9) [37]. Feature 12 is the highest mass in the series, and therefore is possibly the compound fragmented during ionization.

Each chemical feature was compared to the molecular fingerprints of candidate structures. In total, depending on the feature, 151–2757 structures were available to be evaluated for genotoxicity. MLinvitroTox predicted that 0.9–8.6% of the structures would be genotoxic, leaving 4–238 structures per feature (Fig. S11). Of all the structure candidates evaluated for each feature, feature 9, C_14_H_14_O_4_, had the highest proportion that was predicted to be genotoxic (238/2757, 8.6%). However, when focusing only on the top 10% ranked structures, the top features were 11, C_8_H_6_O_3_ (6/31, 19.3%), and 13, C_12_H_10_O_2_ (17/109, 15.7%). For feature 8, C_14_H_13_ClO_4_, which was initially of interest, none of the top 10% of structures was predicted to be genotoxic. Overall, feature 13 had the best match between the MLinvitroTox genotoxicity prediction results for the feature’s MS2 and the top predicted structures. Therefore, it is the highest prioritized feature in zone D.

The 17 candidate structures that were predicted to be genotoxic for feature 13 had similar structural moieties (Fig. S12). These can be generally characterized as substituted naphthalenes and biphenyls. The planar structures of the substituted naphthalenes could be genotoxic through DNA intercalation. Biphenyls can also take planar forms (certain PCBs, for example), possibly explaining that they were predicted to be genotoxic. Feature 12 was predicted to be genotoxic, but only 5.6% of the top candidate structures were active in MLinvitroTox models. The top candidate structure for feature 12, although not predicted to be genotoxic itself, contained similar moieties as predicted for structures linked to feature 13 (Fig. S13A) — adding to the evidence that the features are linked by in-source fragmentation. Two other top structural candidates of feature 12 (Fig. S13B) were predicted to be genotoxic and contained bicyclic structures.

### Conclusions and outlook

This study attempted to identify the chemicals responsible for four genotoxic zones extracted from printed paperboard. We identified one chemical (CMIT) responsible for the strongest genotoxic signal. CMIT is not a newly discovered chemical — we even included it in our mock EDA verification tests — but is not routinely analyzed in food packaging. By using tailored suspect lists, we were able to quickly identify the responsible chemical in one zone as CMIT. It can now be monitored for its prevalence in the market and evaluated for risk to consumers. For another zone, we used machine learning-based toxicity prediction to prioritize a list of candidate features and suggest likely structural moieties, although we did not identify a single responsible chemical. The two remaining genotoxic signals were lost during fractionation so we could not pursue the identities of the responsible chemicals. Further work to refine the fractionation methods and structure elucidation is necessary to identify all chemicals active in HPTLC bioassays. Still, we showed that fractionation with HPTLC substantially reduces chemical features, and that the combined mass spectrometry and bioassay help confirm suspect toxicants. These attributes, and as a sensitive bioassay format, support the further development of HPTLC methods as tools in EDA.

## Supplementary Information

Below is the link to the electronic supplementary material.

Supplementary file1 (DOCX 3665 KB)
