# Supplementary material for: Effect-directed analysis of genotoxicants in food packaging based on HPTLC fractionation, bioassays, and toxicity prediction with machine learning
Source: Anal Bioanal Chem. 2024 Nov 23;417(1):131–42. doi: 10.1007/s00216-024-05632-y (PMC11695491; doi:10.1007/s00216-024-05632-y)
Supplement: Supplementary file 1 — Supplementary file1 (DOCX 3665 KB) [file 216_2024_5632_MOESM1_ESM.docx]

**Supplemental information to**

**Effect-directed analysis of genotoxicants in food packaging based on HPTLC fractionation, bioassays, and toxicity prediction with machine learning**

Alan J. Bergmann,^1^ Katarzyna Arturi,^2^ Andreas Schönborn,^3^ Juliane Hollender,^2,4^ Etiënne Vermeirssen^1^

^1^ Ecotox Center, Eawag, Überlandstrasse 133, 8600 Dübendorf, Switzerland

^2^ Eawag Department of Environmental Chemistry, Überlandstrasse 133, 8600 Dübendorf, Switzerland

^3^ Zurich University of Applied Sciences, Grüental 14, 8820 Wädenswil, Switzerland

^4^ Institute of Biogeochemistry and Pollutant Dynamics, ETH Zürich, 8092 Zürich, Switzerland

**Supplemental Methods**

**Text S1**. Details of HPTLC-umuC method. 2

**Figure S1.** Scheme for one-dimensional fractionation with HPTLC 3

**Text S2.** Optimizing separation of unknown compounds in HPTLC 3

**Figure S2.** Scheme for two-dimensional fractionation with HPTLC 4

**Text S3.** LC-MS optimization 4

**Table S1**. Internal standards used in LC-HRMS evaluations 5

**Table S2.** Compound Discoverer workflow settings 5

**Figure S3.** Settings for molecular formula prediction in SIRIUS 9

**Table S3.** Settings for Fingerprint prediction in SIRIUS 10

**Text S4.** Details of MLinvitroTox modeling for genotoxicity prediction 10

**Table S4.** Model performance of MLinvitroTox genotoxicity prediction 11

**Table S5**. MLinvitroTox results for representative genotoxicants 12

**Supplemental Results**

[**Table S6.** MLinvitroTox results for MS2 of spiked and known native genotoxicants 13](#_Toc180571350)

[**Figure S4.** LC-HRMS features before and after fractionation targeting 4-NQO 13](#_Toc180571351)

[**Figure S5**. MS2 of CMIT standard compared to unknown feature in printed paperboard 13](#_Toc180571352)

[**Figure S6.** Two-dimensional chromatography of printed paperboard extract 14](#_Toc180571353)

[**Figure S7.** Manual extraction of 2D HPTLC zones of printed paperboard with fraction collection 14](#_Toc180571354)

[**Figure S8**. Confirmation of bioactive zones extracted manually with ethyl acetate 15](#_Toc180571355)

[**Text S5.** In source fragmentation 15](#_Toc180571356)

[**Figure S9**. Possible in-source fragmentation of features at 10.6 min prioritized for zone D 16](#_Toc180571357)

[**Figure S10**. Possible in-source fragmentation of features at 15.4 min prioritized for zone D 17](#_Toc180571358)

[**Figure S11**. Ranked candidate structures of features from zone D of printed paperboard with or without predicted genotoxicity 18](#_Toc180571359)

[**Figure S12.** Candidate structures for feature 13 18](#_Toc180571360)

[**Figure S13**. Candidate structures for feature 12 19](#_Toc180571361)

Supplemental Methods

**Text S1**. Details of HPTLC-umuC method.

HPTLC-umuC was performed according to Bergmann et al., 2023.(1) Silica gel 60 HPTLC plates were pre-washed with methanol and dried at 110°C for 0.5 h. Standard chemicals and samples were applied to HPTLC plates in 6 mm bands with an Automated TLC Sampler 4 (ATS4, CAMAG) at 10 mm from plate bottom, at least 20 mm from the sides, and at least 10 mm between the centers of applied bands. Chromatographic development was performed with an Automated Multiple Development 2 (AMD2, CAMAG) as described in the section “EDA strategy”. Atmospheric conditioning solution was 10 mL 25% NH_3_ in 200 mL 18 MΩ water.

Bacteria for the umuC assay, *Salmonella typhimurium* TA1535 psk1002, were obtained from German Collection for Microorganisms and Cell Cultures (DSMZ, Braunschweig, Germany). The evening before a test, one ampule of frozen stock bacteria was thawed and added to 20 mL tryptone-glucose-ampicillin (TGA) media and incubated at 37°C, 220 rpm (Heidolph incubator 1000). After less than 14 h, a morning culture was prepared with a 1:10 dilution of the overnight culture into TGA. After 2 h of incubation, an aliquot of the bacteria morning culture was centrifuged, supernatant removed, and bacteria pellet resuspended in fresh TGA media to 380 ± 20 FAU. A Derivatizer (CAMAG) fitted for 20 x 10 cm HPTLC plates, with red nozzle, at spraying level 6, was used to spray 3 mL bacteria to a prepared HPTLC plate. When 10 x 10 cm or manually cut plates were used for umuC, the plate was placed in the center of the Derivatizer. After a settling period of the bacteria spray, the HPTLC plate was placed in a plastic box pre-heated to 37°C, then placed on a middle shelf in an incubator at 37°C for 2 h. Relative humidity inside each box was maintained at ≥ 90% with two paper towels and 50 mL deionized water. After incubation, the HPTLC plate was dried for approximately 5 min with a hair dryer. The Derivatizer was used with the blue nozzle at spraying level 6 to spray 2 mL of 4-methylumbelliferyl-β-D-galactoside (0.5 mg/mL in B-buffer) onto the HPTLC plate, which was then placed back in the box at 37°C and ≥ 90% relative humidity for 0.5 h. The HPTLC plate was removed and dried again with a hair dryer. Finally, the HPTLC plate was exposed to NH_3_ vapor in a twin trough chamber. Images of HPTLC plates were collected after every step with a Visualizer 2 (CAMAG) with white light and 366 nm illumination. The HPTLC plate tracks were scanned for fluorescence with the Scanner 3 (CAMAG) at 366 nm excitation and 400 nm filter.

**
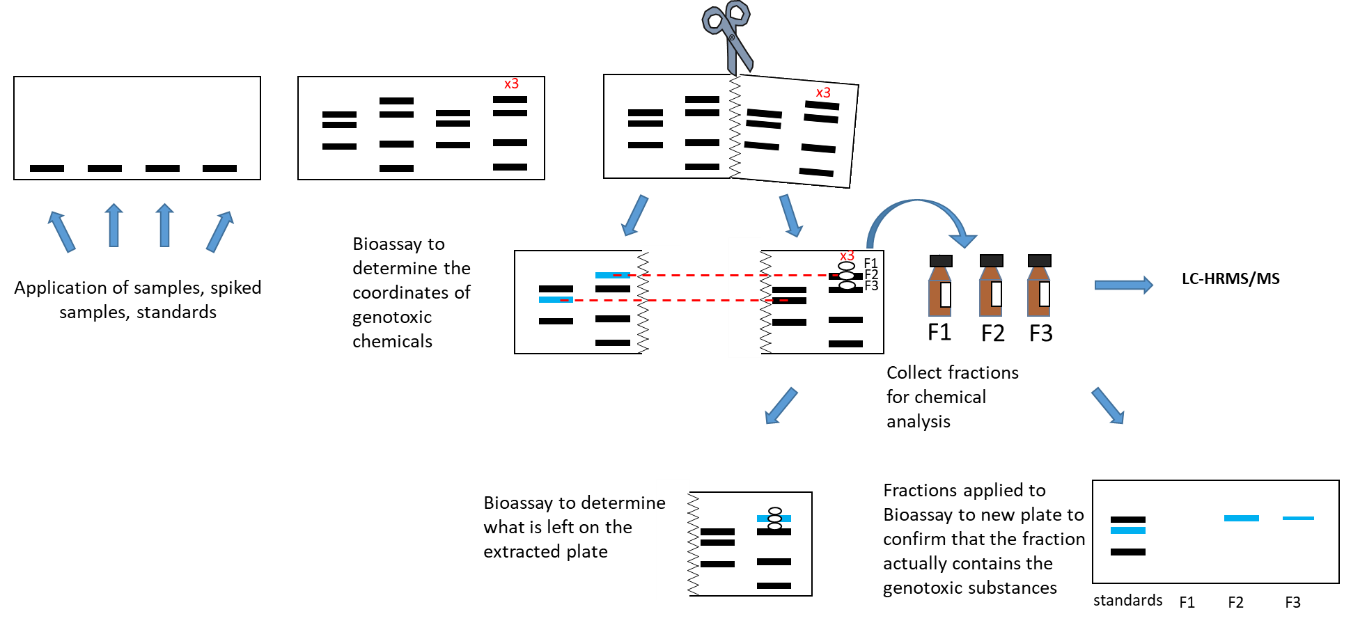
**

**Figure S1.** Scheme for one-dimensional fractionation with HPTLC. The umuC was applied in three steps to determine the Rf of zones of interest, check the remaining sample on the HPTLC plate after elution for chemistry, and confirm that chemicals of interest were captured in fractions from zones of interest.

**Text S2.** Optimizing separation of unknown compounds in HPTLC

We began with a quick and simple screening method with a focusing step with 100% methanol to 20 mm, then 50:50 acetone:n-hexane to 80 mm. Multiple genotoxic zones were apparent in paperboard extracts. Two zones were especially strong in printed paperboard at Rfs 0.6-0.7 (Figure 1B). More intricate chromatography followed, which consisted of a focusing step with 100% methanol to 20 mm, then separation with 100% acetone to 30 mm, 75:25 acetone:ethyl acetate to 40 mm, 100% ethyl acetate to 50 mm, 66.6:33.4 ethyl acetate:n-hexane to 60 mm, and 50:50 ethyl acetate:n-hexane to 80 mm. This method revealed that there were, in fact, at least four different genotoxic zones appearing in the ethyl acetate-based section of the track. To improve resolution between bioactive zones, the chromatography method was simplified to focus on the ethyl acetate zones. The final optimized chromatography consisted of a focusing step with 100% methanol to 20 mm, then separation with 100% ethyl acetate to 35 mm, 66.6:33.4 ethyl acetate:n-hexane to 55 mm, and 50:50 ethyl acetate:n-hexane to 80 mm.


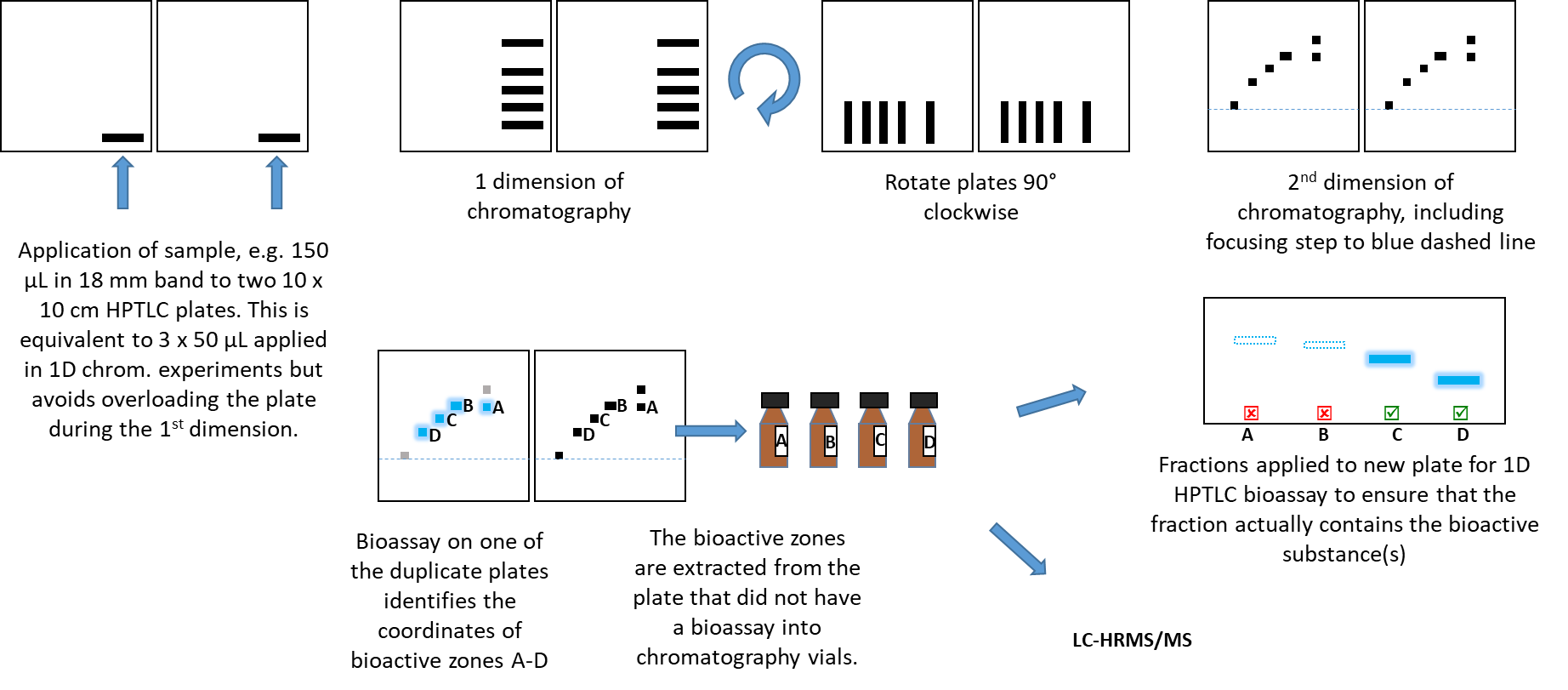


**Figure S2.** Scheme for two-dimensional fractionation with HPTLC. Parallel 10 x 10 cm HPTLC plates were used to chromatographically develop a paperboard extract first in one direction. Then the plates were rotated 90° clockwise and developed in a second, perpendicular, direction with different solvent mixtures. One plate was used to determine the coordinates of genotoxic zones by applying the umuC bioassay. The genotoxic bands were then collected from the plate into glass vials for subsequent analysis.

**Text S3.** LC-MS optimization.

We prepared for chemical analysis by evaluating the response of several standard chemicals (listed in Table S4) representing known genotoxicants and chemicals related to food packaging. We evaluated the responses in positive and negative mode electrospray ionization, and using a neutral mobile phase. We selected conditions that detected most of these representative chemicals, recognizing that unknown toxicants could have different optimal parameters. The final settings for data acquisition are given in the main text.

**Table S1**. Internal standards used in LC-HRMS evaluations, based on subset from previous work in similar analytical conditions.(2, 3)

| Standard | Formula | M+H | Exact Mass | Retention time | LogKow (XLogP3-AA, Pubchem) |
| --- | --- | --- | --- | --- | --- |
| Guanyl-Urea-15N4 | C2H6[15]N4O | 107.0496 | 106.0423 | 2.61 | -1.4 |
| Metformin-D6 | C4H5N5D6 | 136.1464 | 135.1391 | 3.00 | -1.3 |
| Gemcitabine-13C,15N2 | C8H11F2NO4[13]C[15]N2 | 267.0765 | 266.0692 | 6.84 | -1.5 |
| Paracetamol-D4 (3-Acetamidophenol-d4) | C8H5D4N1O2 | 156.0952 | 155.0879 | 10.11 | 0.7 |
| Sulfathiazol-D4 | C9H5D4N3O2S2 | 260.0455 | 259.0382 | 10.20 | 0.1 |
| Benzotriazol-D4 | C6H1D4N3 | 124.0802 | 123.0729 | 13.08 | 1.4 |
| Clozapine-D8 | C18H11D8ClN4 | 335.1873 | 334.18 | 13.89 | 3.1 |
| Mesotrion D3 | C14H10D3NO7S | 343.0680 | 342.0607 | 15.63 | 0.7 |
| Clarithromycin-D3 | C38H66D3NO13 | 751.5025 | 750.4952 | 16.38 | 3.2 |
| Bezafibrat-D4 | C19H16D4ClNO4 | 366.1399 | 365.1326 | 18.97 | 3.8 |
| Fenofibrate-D6 | C20H15D6ClO4 | 367.1584 | 366.1511 | 21.61 | 5.2 |
| Tipranavir-d4 | C31H29D4F3N2O5S | 607.2386 | 606.2313 | 20.61 | 7 |

**Table S2.** Compound Discoverer workflow settings

Processing node 1: Select Spectra

1. Spectrum Properties Filter:

- Lower RT Limit: 0

- Upper RT Limit: 0

- First Scan: 0

- Last Scan: 0

- Ignore Specified Scans: (not specified)

- Lowest Charge State: 0

- Highest Charge State: 0

- Min. Precursor Mass: 100 Da

- Max. Precursor Mass: 5000 Da

- Total Intensity Threshold: 0

- Minimum Peak Count: 1

2. Scan Event Filters:

- Mass Analyzer: (not specified)

- MS Order: Any

- Activation Type: (not specified)

- Min. Collision Energy: 0

- Max. Collision Energy: 1000

- Scan Type: Any

- Polarity Mode: Is +

3. Peak Filters:

- S/N Threshold (FT-only): 1.5

4. Replacements for Unrecognized Properties:

- Unrecognized Charge Replacements: 1

- Unrecognized Mass Analyzer Replacements: ITMS

- Unrecognized MS Order Replacements: MS2

- Unrecognized Activation Type Replacements: CID

- Unrecognized Polarity Replacements: +

- Unrecognized MS Resolution@200 Replacements: 60000

- Unrecognized MSn Resolution@200 Replacements: 30000

5. General Settings:

- Precursor Selection: Use MS(n - 1) Precursor

- Use Isotope Pattern in Precursor Reevaluation: True

- Provide Profile Spectra: Automatic

- Store Chromatograms: False

Processing node 33: Align Retention Times

1. General Settings:

- Alignment Model: Adaptive curve

- Alignment Fallback: Use Linear Model

- Maximum Shift [min]: 2

- Shift Reference File: True

- Mass Tolerance: 5 ppm

- Remove Outlier: True

Processing node 45: Detect Compounds

1. General Settings:

- Mass Tolerance [ppm]: 2 ppm

- Intensity Tolerance [%]: 30

- S/N Threshold: 3

- Min. Peak Intensity: 100000

- Ions: [M+H]+1; [M+K]+1; [M+Na]+1; [M+NH4]+1; [M-H]-1

- Base Ions: [M+H]+1; [M-H]-1

- Min. Element Counts: C H

- Max. Element Counts: C90 H190 Br3 Cl4 K2 N10 Na2 O18 P3 S5

2. Peak Detection:

- Filter Peaks: True

- Max. Peak Width [min]: 0.5

- Remove Singlets: True

- Min. # Scans per Peak: 5

- Min. # Isotopes: 2

3. Isotope Grouping:

- Min. Spectral Distance Score: 0

- Remove Potentially False Positive Isotopes: True

Processing node 24: Group Compounds

1. Compound Consolidation:

- Mass Tolerance: 2 ppm

- RT Tolerance [min]: 0.1

2. Fragment Data Selection:

- Preferred Ions: [M+H]+1; [M-H]-1

Processing node 36: Fill Gaps

1. General Settings:

- Mass Tolerance: 2 ppm

- S/N Threshold: 3

- Use Real Peak Detection: True

Processing node 37: Mark Background Compounds

1. General Settings:

- Max. Sample/Blank: 5

- Max. Blank/Sample: 0

- Hide Background: True

Processing node 22: Search ChemSpider

1. Search Settings:

- Database(s):

ACToR: Aggregated Computational Toxicology Resource

Aurora Fine Chemicals

DISMA, Department of Agri-Food Molecular Sciences, University of Milano, Italy

EPA DSSTox

EPA Toxcast

Food and Agriculture Organization of the United Nations

FooDB

- Search Mode: By Formula or Mass

- Mass Tolerance: 2 ppm

- Max. # of results per compound: 10

- Max. # of Predicted Compositions to be searched per Compound: 1

- Result Order (for Max. # of results per compound): Order By Reference Count (DESC)

2. Predicted Composition Annotation:

- Check All Predicted Compositions: True

Processing node 40: Apply mzLogic

1. Search Settings:

- FT Fragment Mass Tolerance: 10 ppm

- IT Fragment Mass Tolerance: 0.4 Da

- Max. # Compounds: 0

- Max. # mzCloud Similarity Results to consider per Compound: 10

- Match Factor Threshold: 30

Processing node 23: Search Mass Lists

1. Search Settings:

- Mass Lists: GenotoxCustomInclusionList_searchList.massList

- Mass Tolerance: 2 ppm

- Use Retention Time: False

- RT Tolerance [min]: 0.25

Processing node 26: Predict Compositions

1. Prediction Settings:

- Mass Tolerance: 2 ppm

- Min. Element Counts: C H

- Max. Element Counts: C90 H190 Br3 Cl4 F6 N10 O18 P3 S5

- Min. RDBE: -5

- Max. RDBE: 40

- Min. H/C: 0.1

- Max. H/C: 3.5

- Max. # Candidates: 10

- Max. # Internal Candidates: 200

2. Pattern Matching:

- Intensity Tolerance [%]: 30

- Intensity Threshold [%]: 0.1

- S/N Threshold: 3

- Min. Spectral Fit [%]: 30

- Min. Pattern Cov. [%]: 80

- Use Dynamic Recalibration: True

3. Fragments Matching:

- Use Fragments Matching: True

- Mass Tolerance: 4 ppm

- S/N Threshold: 3

Processing node 29: Assign Compound Annotations

1. General Settings:

- Mass Tolerance: 5 ppm

2. Data Sources:

- Data Source #1: mzCloud Search

- Data Source #2: Predicted Compositions

- Data Source #3: MassList Search

- Data Source #4: ChemSpider Search

- Data Source #5: (not specified)

- Data Source #6: (not specified)

- Data Source #7: (not specified)

3. Scoring Rules:

- Use mzLogic: True

- Use Spectral Distance: True

- SFit Threshold: 20

- SFit Range: 20

Processing node 27: Search mzCloud

1. General Settings:

- Compound Classes: All

- Precursor Mass Tolerance: 10 ppm

- FT Fragment Mass Tolerance: 10 ppm

- IT Fragment Mass Tolerance: 0.4 Da

- Library: Autoprocessed; Reference

- Post Processing: Recalibrated

- Max. # Results: 20

- Annotate Matching Fragments: False

2. DDA Search:

- Identity Search: HighChem HighRes

- Match Activation Type: True

- Match Activation Energy: Match with Tolerance

- Activation Energy Tolerance: 20

- Apply Intensity Threshold: True

- Similarity Search: Similarity Forward

- Match Factor Threshold: 60

3. DIA Search:

- Use DIA Scans for Search: False

- Max. Isolation Width [Da]: 500

- Match Activation Type: False

- Match Activation Energy: Any

- Activation Energy Tolerance: 100

- Apply Intensity Threshold: False

- Match Factor Threshold: 20

Processing node 14: Merge Features

1. Peak Consolidation:

- Mass Tolerance: 5 ppm

- RT Tolerance [min]: 0.1

Processing node 39: Differential Analysis

1. General Settings:

- Log10 Transform Values: True


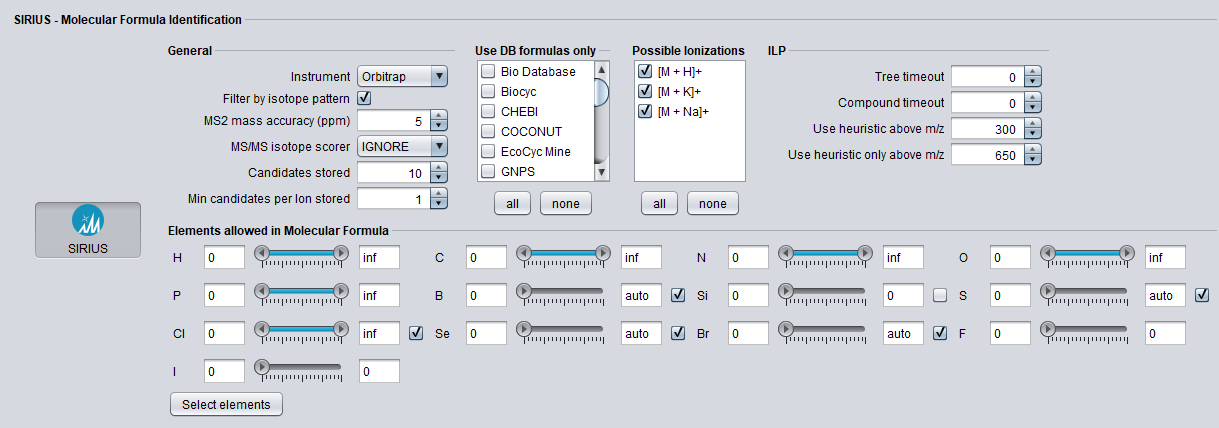


**Figure S3.** Settings for molecular formula prediction in SIRIUS.

**Table S3.** Settings for Fingerprint prediction in SIRIUS.

| possible adducts | databases searched | |
| --- | --- | --- |
| [M+H]+  [M+K]+  [M+Na]+ | Bio Database  Biocyc  CHEBI  COCONUT  EcoCyc Mine  GNPS  HMDB  HSDB  KEGG  KEGG Mine  KNApSAcK | Maconda  MeSH  NORMAN  Natural Products  Plantcyc  PubChem  PubMed  YMDB  YMDB Mine  ZINC bio |

**Text S4.** Details of MLinvitroTox modeling for genotoxicity prediction

Molecular fingerprints and toxicity records were combined creating input for the training of supervised MLinvitroTox classifiers for prediction of genotoxicity. For each input data set, the low-variability and highly-correlating features were filtered off, decreasing the number of input features to approximately 350-400, depending on the endpoint.

For the training of the data, a subset of CSI:FingerID molecular toxicity fingerprints with 2363 bits (openbabel fp3, openbabel fp4, MACCS, PubChem, Klekkota, and custom smarts and ring formulations) were generated for cleaned-up structures from DSSTox database as described previously.(4)

For p53 activation, that the final hitcall per target/chemical pair was established by majority voting (active ≥ 0.50) of the endpoints belonging to the mechanistic target. If more than 50% of the hitcalls were positive (toxic or 1), the consensus hitcall was designated as positive; otherwise, it was designated as negative (0). For DNA damage, the final hitcall was “active” when the LC_50_ for either DT40_100 or DT40_657 was at least 3 times smaller than the corresponding (for the same tested chemical) LC_50_ for DT40_wt. We used the AC50 estimates generated by the tcpl package for both the control assay (DT40_wt) and the target assays (DT40_100 and DT40_657). AC50 ratios were calculated between each target and control, and custom hitcalls were assigned as follows: a hitcall of 1 was assigned only if the AC50 for the target assay was three times lower than the AC50 for the control assay; otherwise, an inactive (0) hitcall was assigned. For the purposes of calculations, the original inactive (0) hitcalls were assigned an AC50 of 1000 µM.

XGBoost classifers were trained with autoML functionality in KNIME.(5) The compounds present in the genotoxic suspect list were removed from the data prior to modeling and used as an external validation of performance. The models were trained with 0.85/0.15 train/test split with SMOTE oversampling on the train data and hyperparameter-tuning (random search of max_depth [5-25, step_size = 5] and eta [0.01-0.5, step_size = 0.05], min_iterations = 20, and early stopping after 10) nested into 5-fold cross-validation (CV=5). For both cross-validation as well as testing, F1-measure was used as an evaluation metric. The models were saved and applied subsequently on (1) the molecular fingerprints computed for the compounds on the genotoxic list, (2) the molecular fingerprints for the prioritized features derived from SIRIUS, and (3) lists of candidates for the prioritized features derived from SIRIUS. The binary predictions for each Mechanistic Target/chemical feature pair: 0 (inactive) or 1 (active) was based on the default 0.5 threshold.

**Table S4.** Model performance of MLinvitroTox genotoxicity prediction

|  | DT40ratioup | p53BLAup |
| --- | --- | --- |
| Endpoint | differential cytotoxicity as indication of DNA damage | p53 induction as indication of DNA binding |
| # entries in dataset | 3057 | 5953 |
| Model | XGBoost Trees | XGBoost Trees |
| extracted_params | eta = 0.2, Max_depth = 10 | eta = 0.2, Max_depth = 10 |
| Accuracy | 0.957 | 0.961 |
| Cohen's kappa | 0.915 | 0.922 |
| Recall | 1 | 0.993 |
| Precision | 0.921 | 0.933 |
| F-measure | 0.959 | 0.962 |
| Static Prediction | NO | NO |

**Table S5**. MLinvitroTox results for representative genotoxicants.

| **Chemical name** | **CAS** | **Source for genotoxicity** | **TOX21DT40ratioup** | **TOX21p53BLAup** | **Final hitcall** |
| --- | --- | --- | --- | --- | --- |
| 5-Chloro-2-methyl-3(2H)-isothiazolone | 26172–55–4 | Bergmann et al. 2023(1) | 0.990 | 0.478 | 1 |
| Etoposide | 33419–42–0 | Bergmann et al. 2023(1) | 0.991 | 0.994 | 1 |
| Nalidixic acid | 389–08–2 | Bergmann et al. 2023(1) | 0.002 | 0.007 | 0 |
| Mitomycin C | 50–07–7 | Bergmann et al. 2023(1) | 0.046 | 0.977 | 1 |
| 4-Nitroquinoline-1-oxide | 56–57–5 | Bergmann et al. 2023(1) | 0.723 | 0.900 | 1 |
| Nitrofurantoin | 67–20–9 | Bergmann et al. 2023(1) | 0.024 | 0.257 | 0 |
| 4,4'-Methylenebis(2-chloroaniline) | 101-14-4 | Kuslikis et al. 1991(6) | 0.002 | 0.895 | 1 |
| 4,4'-Oxydianiline | 101-80-4 | Van Bossuyt et al. 2019(7) | 0.006 | 0.480 | 0 |
| 1,3-Benzenediamine | 108-45-2 | ECHA registration dossier(8) | 0.614 | 0.128 | 1 |
| 3,3'-Dimethylbenzidine | 119-93-7 | Morgan et al. 1991(9) | 0.004 | 0.905 | 1 |
| 2,6-Toluenediamine | 823-40-5 | Reifferscheid and Heil 1996(10) | 0.930 | 0.188 | 1 |
| Michler's ketone | 90-94-8 | Ozaki 2004(11) | 0.002 | 0.941 | 1 |
| 2,4-Diaminotoluene | 95-80-7 | Séverin et al. 2005,(12) Reifferscheid and Heil 1996(10) | 0.072 | 0.714 | 1 |

Supplemental Results

**Table S6.** MLinvitroTox results for MS2 of spiked and known native genotoxicants.

| Chemical | Spiked or native | Experiment description | Genotoxicity prediction results | |
| --- | --- | --- | --- | --- |
|  |  |  | TOX21DT40ratioup | TOX21p53BLAup |
| CMIT | spiked | 1D, TLC-MS interface, MeOH | 0.548 | 0.863 |
| CMIT | native | 1D, TLC-MS interface, methanol | 0.548 | 0.871 |
| CMIT | native | 2D, manually, ethyl acetate | 0.548 | 0.871 |
| CMIT | native | 2D, manual, methanol | 0.548 | 0.871 |
| 4-NQO | spiked | 2D, manual, methanol | 0.059 | 0.889 |


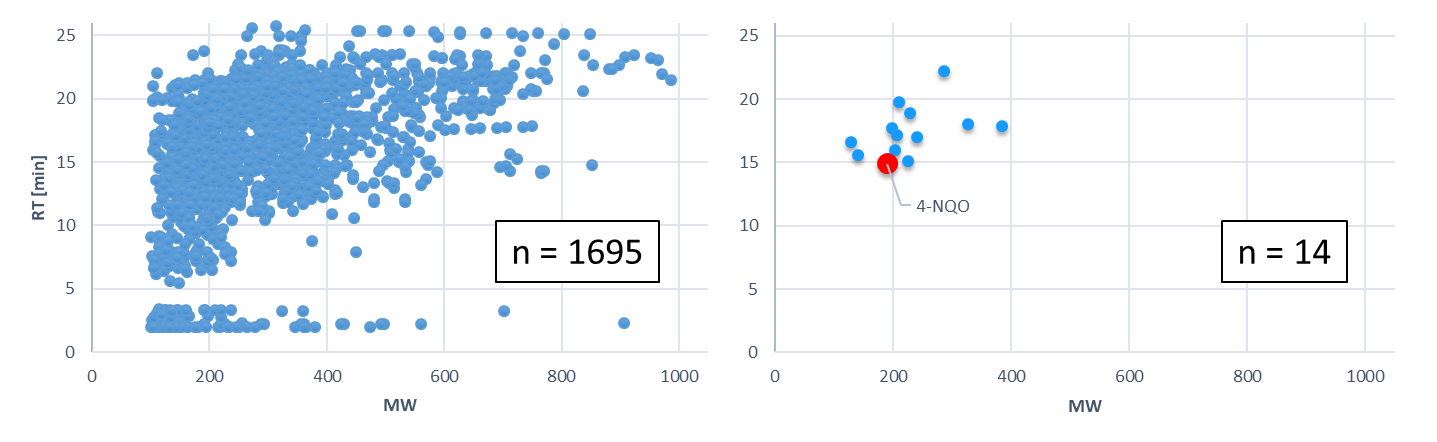


**Figure S4.** LC-HRMS features before and after fractionation targeting 4-NQO. Chemical features in a parent recycled paperboard spiked with 4-NQO and CMIT (left) and the bioactive fraction that corresponded to 4-NQO (right). The number of features was reduced from 1695 to 14 and 4-NQO was successfully measured and prioritized.


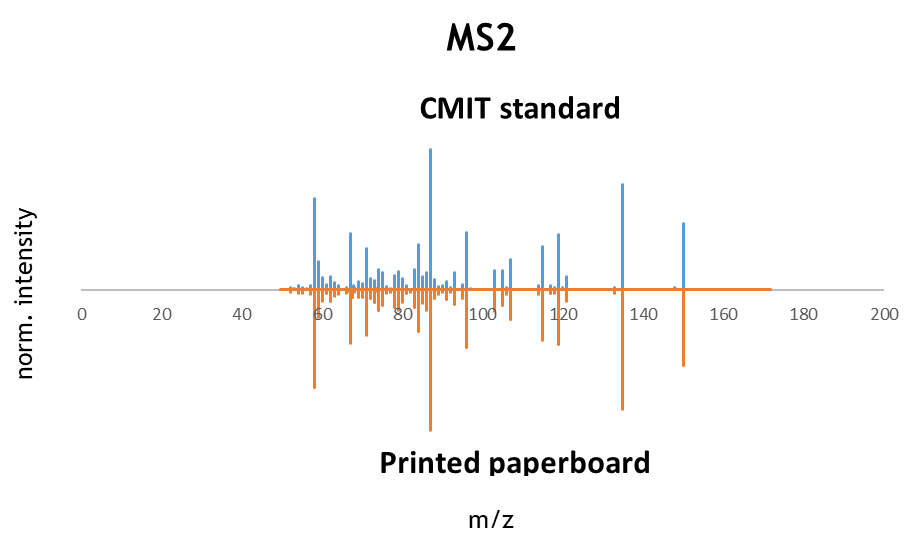


**Figure S5**. MS2 of CMIT standard compared to unknown feature in printed paperboard.


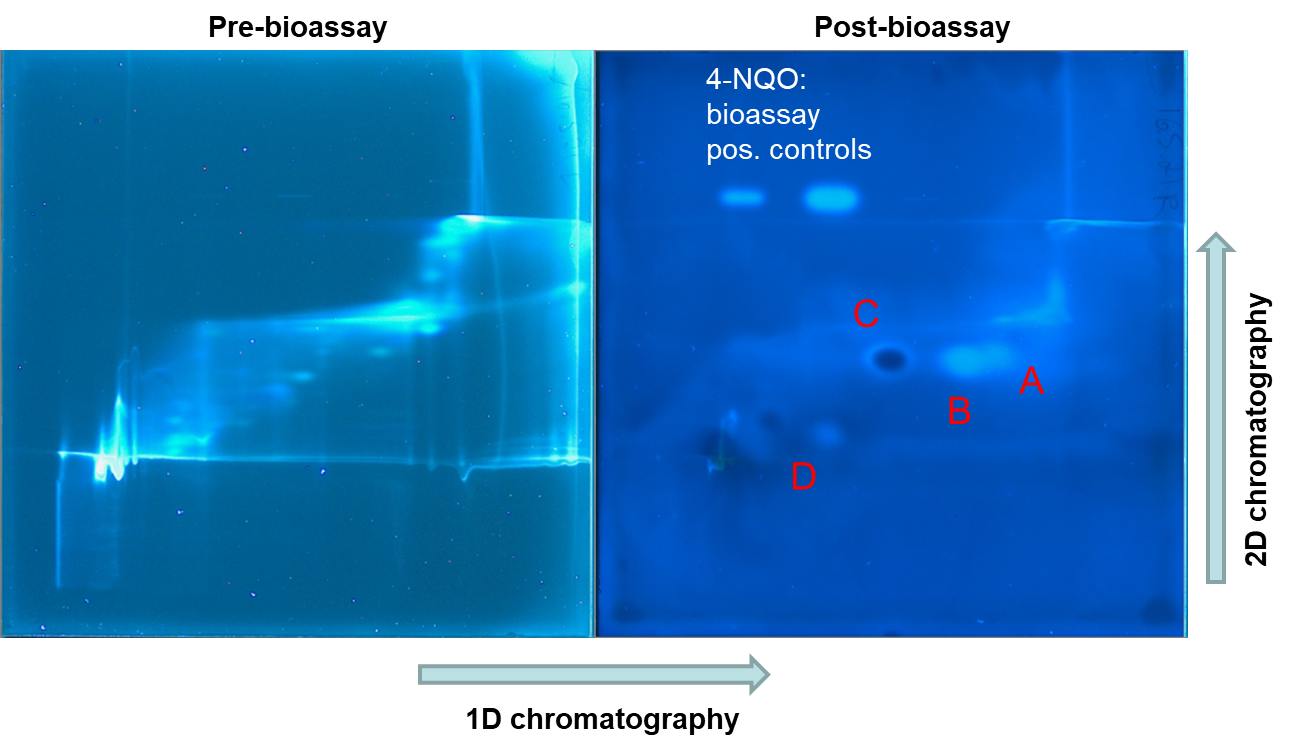


**Figure S6.** Two-dimensional chromatography of printed paperboard extract. Zones A, B, C, and D were further separated from natively fluorescent compounds. Positive controls, 4-NQO at 0.31 and 2.5 ng were applied to the plate after chromatography steps were completed. For manual fraction collection, 4-NQO at 2.5 ng was eluted as a fraction collection control.


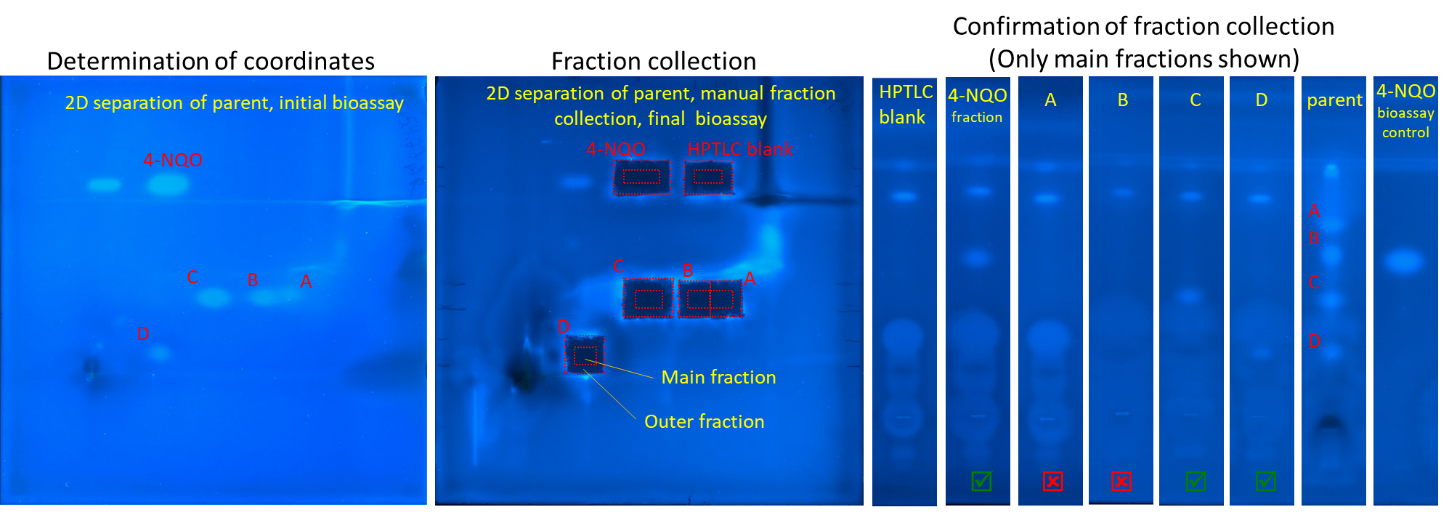


**Figure S7.** Manual extraction of 2D HPTLC zones of printed paperboard with fraction collection. This figure corresponds to Figure 2. A central, “main”, zone was removed and extracted separately from the surrounding, “outer”, silica. The process was applied to the 4-NQO positive control and a blank zone, “HPTLC blank”.


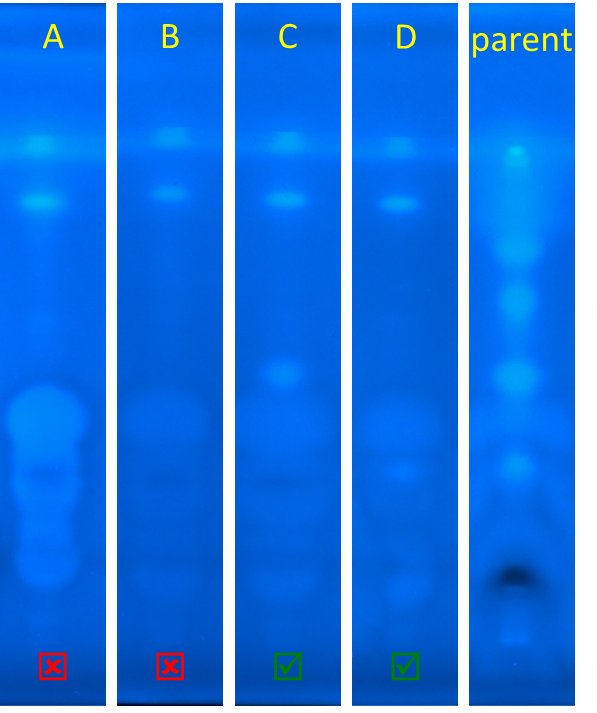


**Figure S8**. Confirmation of bioactive zones extracted manually with ethyl acetate. Fractions were reapplied in one dimensional HPTLC-umuC. Fractions A and B were not confirmed (red X) while fractions of zones C and D were confirmed to contain the bioactive chemicals (green check). Parent (unfractionated) printed paperboard extract was analyzed again on the same plate and is shown as a reference for the zone retention factors.

**Text S5.** In-source fragmentation.

Feature 13 (m/z 187.0754) is a major MS2 fragment of ion 247.0967, which is in turn a major fragment of feature 12 (m/z 406.1861), all at RT 15.39 (See Figure S9). 247.0967 is not a prioritized feature, although it is one of the most abundant features in the parent sample that is also observed in the 2D methanol fraction. 247.0967 was excluded from the prioritized feature list because it is present at lower than expected ratios in the ethyl acetate fraction compared to the parent sample. If these features are present due to in-source fragmentation, perhaps degree of in-source fragmentation is influenced by matrix and therefore different in parent samples compared to the “cleaner” fractions, resulting in warping the apparent fraction-parent ratios.


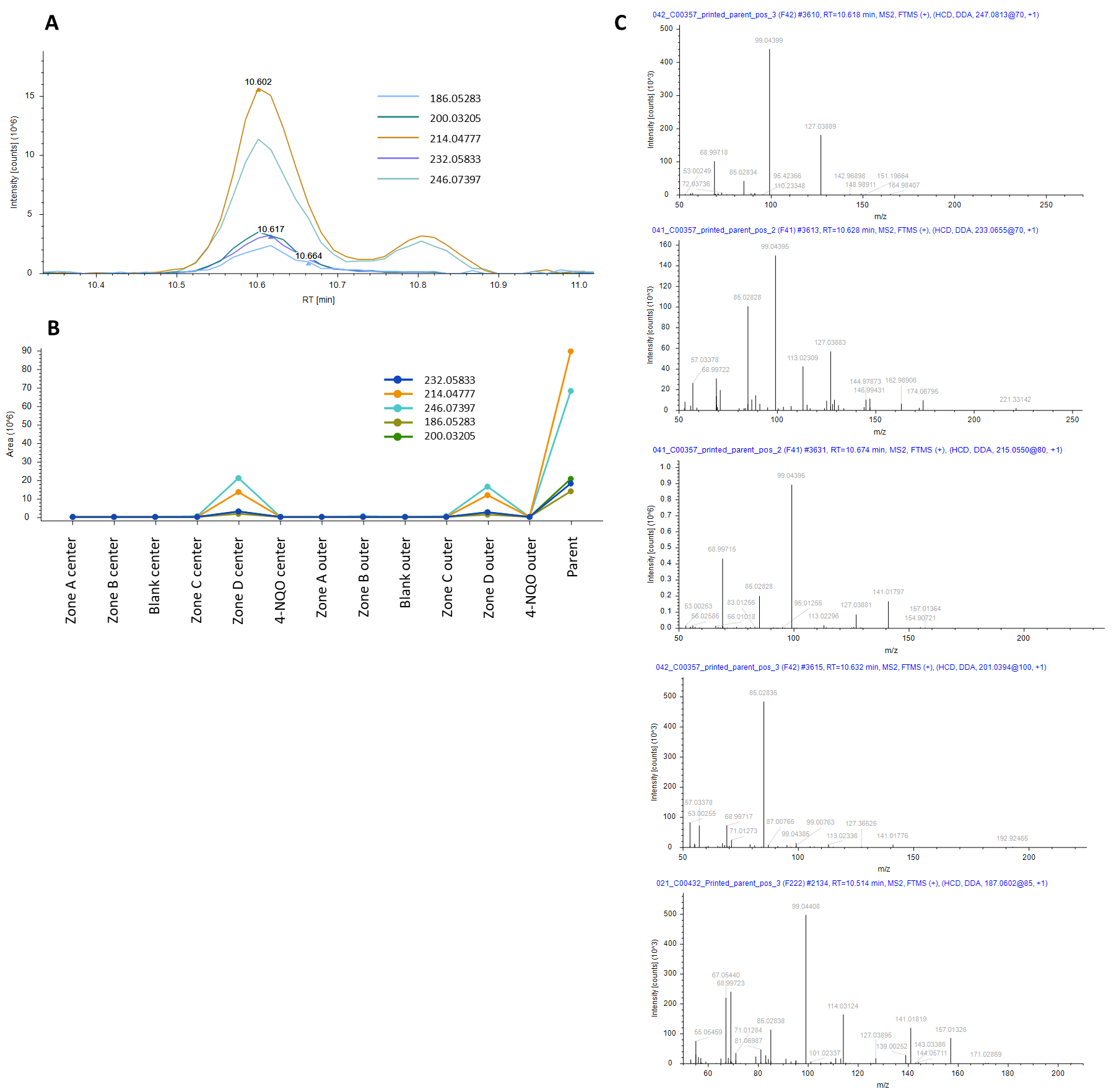


**Figure S9**. Possible in-source fragmentation of features at 10.6 min prioritized for zone D. Representative data from 2D fractionation using manual extraction with methanol, corresponding to table 1, column 4. (A) Overlaid extracted ion chromatograms of the MS1 traces. Traces are labeled with the calculated molecular weight. (B) Abundances of features among fractions and parent sample. The features are labeled with the calculated molecular weight. (C) MS2 spectra of each feature suspected of being associate with in-source ionization. MS2 for 186.0528 was collected in a follow-up measurement campaign so the RT is shifted.


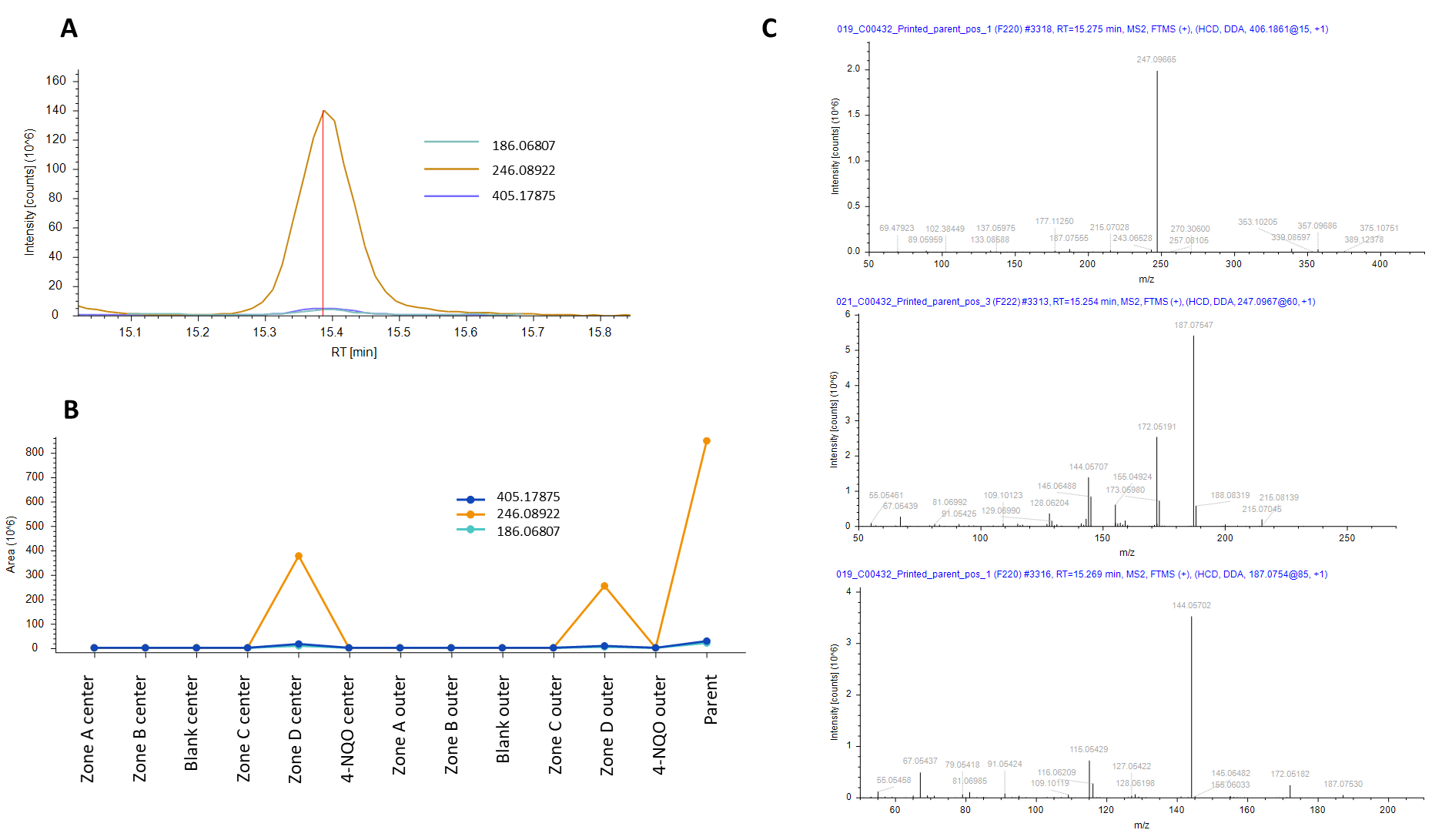


**Figure S10**. Possible in-source fragmentation of features at 15.4 min prioritized for zone D.


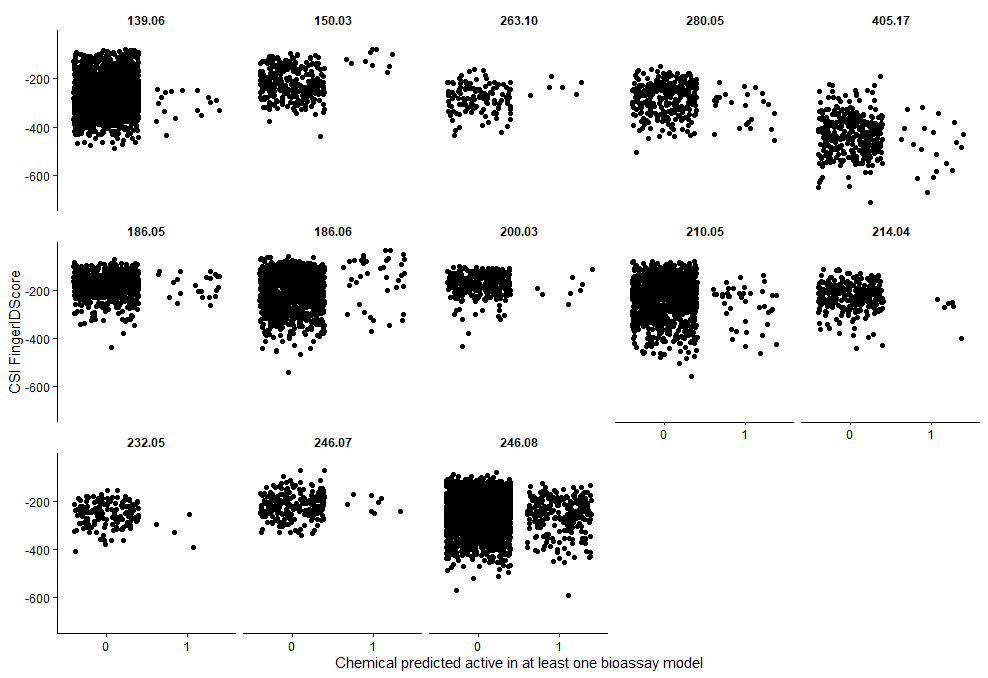


**Figure S11**. Ranked candidate structures of features from zone D of printed paperboard with or without predicted genotoxicity. Black points represent all of the considered chemical structures ranked by the similarity of their chemical fingerprints to the fingerprints derived from the MS2 of the unknown chemical features (CSI FingerIDScore). The chemicals predicted to be genotoxic (“1”, i.e. >50% probability in at least one of two genotoxicity bioassay models) are displayed separately from those not predicted to be genotoxic (“0”).


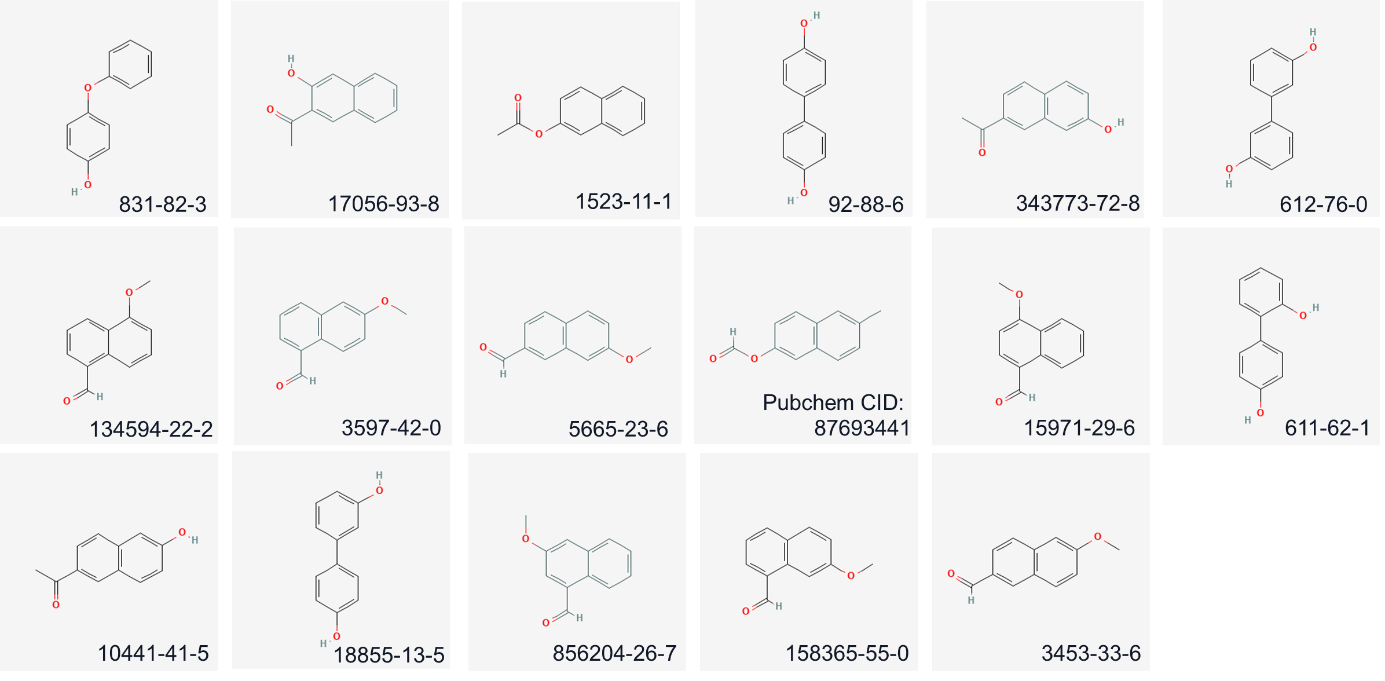
**Figure S12.** Candidate structures for feature 13. Candidates were prioritized by being (1) in the top 10% of ranked structures compared to MS2 of the unknown and (2) predicted to be genotoxic in at least one bioassay. Source of structures: <https://pubchem.ncbi.nlm.nih.gov/>. Structures are shown with CAS number when possible, or the PubChem chemical identifier (PubChem CID) when not.


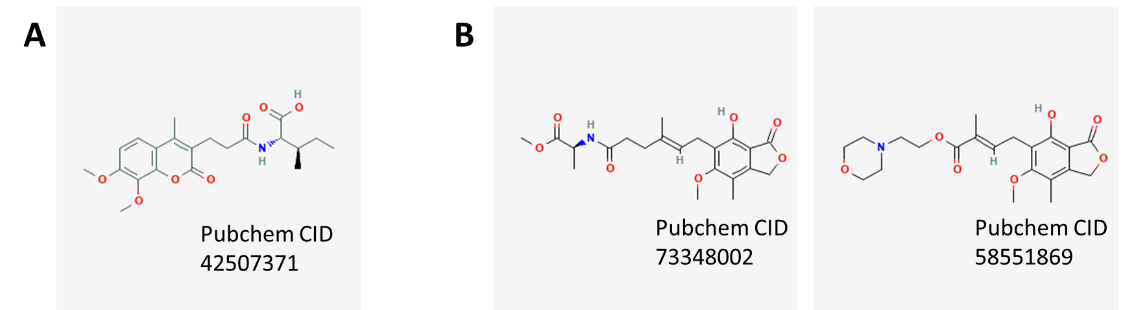


**Figure S13.** Candidate structures for feature 12. (A) Top structural candidate according to SIRIUS CSIFingerID Score but not predicted to be genotoxic. (B) Two structures in the top 10% that were predicted to be genotoxic in at least one bioassay. PubChem chemical identifier (Pubchem CID) given for compounds without CAS numbers. One stereoisomer is given as an example.

References

1. Bergmann AJ, Breitenbach M, Muñoz C, Simon E, McCombie G, Biedermann M, et al. Towards detecting genotoxic chemicals in food packaging at thresholds of toxicological concern using bioassays with high-performance thin-layer chromatography. Food Packaging and Shelf Life. 2023;36:101052.

2. Kiefer K, Muller A, Singer H, Hollender J. New relevant pesticide transformation products in groundwater detected using target and suspect screening for agricultural and urban micropollutants with LC-HRMS. Water Research. 2019;165:114972.

3. Mechelke J, Longrée P, Singer H, Hollender J. Vacuum-assisted evaporative concentration combined with LC-HRMS/MS for ultra-trace-level screening of organic micropollutants in environmental water samples. Analytical and Bioanalytical Chemistry. 2019;411(12):2555-67.

4. Arturi K, Hollender J. Machine Learning-Based Hazard-Driven Prioritization of Features in Nontarget Screening of Environmental High-Resolution Mass Spectrometry Data. Environmental science & technology. 2023.

5. Garmpis S, Maragoudakis M, Garmpis A. Assisting Educational Analytics with AutoML Functionalities. Computers. 2022;11(6):97.

6. Kuslikis BI, Trosko JE, Braselton WE, Jr. Mutagenicity and effect on gap - junctional intercellular communication of 4, 4′-methylenebis(2-chloroaniline) and its oxidized metabolites. Mutagenesis. 1991;6(1):19-24.

7. Van Bossuyt M, Van Hoeck E, Vanhaecke T, Rogiers V, Mertens B. Prioritizing substances of genotoxic concern for in-depth safety evaluation using non-animal approaches: The example of food contact materials. ALTEX. 2019;36(2):215-30.

8. Registration Dossier: m-phenylenediamine [Internet]. [cited 05.06.2024]. Available from: <https://echa.europa.eu/de/registration-dossier/-/registered-dossier/14386/7/7/2>.

9. MORGAN DL, JOKINEN MP, HASEMAN IJK, ULLAND IBM, PARKER GA, LEMEN JK, et al. Carcinogenicity of 3,3 ’ -Dimethylbenzidine Dihydrochloride Given in Drinlung Water to F344/N Rats. JOURNAL OF THE AMERICAN COLLEGE OF TOXICOLOGY. 1991;10(2):255-66.

10. Reifferscheid G, Heil J. Validation of the SOS/umu test using test results of 486 chemicals and comparison with the Ames test and carcinogenicity data. Mutation Research/Genetic Toxicology. 1996;369(3-4):129-45.

11. Ozaki A, Yamaguchi Y, Fujita T, Kuroda K, Endo G. Chemical analysis and genotoxicological safety assessment of paper and paperboard used for food packaging. Food and chemical toxicology : an international journal published for the British Industrial Biological Research Association. 2004;42(8):1323-37.

12. Séverin I, Jondeau A, Dahbi L, Chagnon MC. 2,4-Diaminotoluene (2,4-DAT)-induced DNA damage, DNA repair and micronucleus formation in the human hepatoma cell line HepG2. Toxicology. 2005;213(1-2):138-46.
